# Structural and functional analysis reveals the catalytic mechanism and substrate binding mode of the broad-spectrum endolysin Ply2741

**DOI:** 10.1080/21505594.2024.2449025

**Published:** 2025-01-14

**Authors:** Shuang Wang, Xinxin Li, Jiahui Ma, Xiaochao Duan, Haiyan Wang, Linkang Wang, Dayue Hu, Wenwu Jiang, Xiangmin Li, Ping Qian

**Affiliations:** a National Key Laboratory of Agricultural Microbiology, Hubei Hongshan Laboratory, Huazhong Agricultural University, Wuhan, China; b The Cooperative Innovation Centre for Sustainable Pig Production, Huazhong Agricultural University, Wuhan, China; c College of Veterinary Medicine, Huazhong Agricultural University, Wuhan, China

**Keywords:** Antibiotic resistance, phage endolysins, antimicrobial agents, lytic activity, structure

## Abstract

The emergence of antibiotic-resistant bacteria has attracted interest in the field of endolysins. Here, we analyzed the diversity of *Streptococcus* endolysins and identified a new endolysin, Ply2741, that exhibited broad-spectrum bactericidal activity. Our results demonstrated that Ply2741 could effectively eradicate multidrug-resistant gram-positive pathogens *in vitro* and *in vivo*. Structural analysis revealed that the bactericidal activity of Ply2741 depends on the classic “Cys-His-Asn” catalytic triad. Site-directed mutagenesis results further identified that the conserved residue Gln29, located near the catalytic triad, also contributes to the lytic activity of Ply2741. Furthermore, the key residues (R189 and W250) in the Ply2741 cell wall binding domain (CBD) responsible for binding to peptidoglycan were revealed by molecular docking and fluorescence-activated cell sorting (FACS) analysis. Ply2741 demonstrates a broad lytic spectrum, with significant bactericidal activity against *Enterococcus*, *Staphylococcu*s, *and Streptococcus* and species. To the best of our knowledge, we found that residue Gln29 participated in the lytic activity of endolysin for the first time. Additionally, we systematically elucidate the binding mode and key residues of the Ply2741CBD. This study proposes Ply2741 as a potential antibiotic substitute and provides a structural basis for the modification and design of endolysins.

## Introduction

Several species of *Streptococcus*, *Staphylococcus*, and *Enterococcus* are commensal microbiota that are widely present in humans [1]. However, certain species within these genera can cause serious infections, thereby posing a significant risk to public health. *Group B Streptococcus* (GBS) is an opportunistic pathogen that can cause neonatal pneumonia, septicemia, and meningitis [2]. *Streptococcus suis*, initially recognized as the predominant pathogen in swine, has gradually emerged as an important concern for global public health safety [3]. Palmieri et al. reported that *S. suis* has become an antibiotic-resistant reservoir [4]. *Staphylococcus aureus* and *Enterococcus* are important pathogens in community-acquired and hospital infection [5,6]. *S. aureus* can cause blood and respiratory infections and even septicemia [7]. The prevalence of *Methicillin-resistant Staphylococcus aureus* (MRSA) has increased significantly in communities and hospitals in various countries [8–10]. *Enterococcus* is a common cause of endocarditis and contributes to a series of infections in community environments [11]. The World Health Organization (WHO) has placed vancomycin-resistant *Enterococcus* (VRE) at the top of the global priority list of drug-resistant bacteria [12]. The continuing emergence of antimicrobial resistance (AMR) has been identified as a major threat to humanity by WHO [13]. With the continued emergence of multidrug-resistant bacteria and the slow progress in the development of new antibiotics, innovative therapeutics are urgently needed to combat bacterial infections.

Bacteriophages and derived enzymes are potential therapeutic agents against antibiotic-resistant bacteria [14]. However, phage therapeutics present significant challenges such as the development of bacterial resistance, immunogenicity, and horizontal gene transfer [15–17]. In contrast, endolysins, which are hydrolases produced by bacteriophages, exhibit independent antimicrobial activity, a wider lytic spectrum, and a lower likelihood of bacterial resistance development [18]. Several studies have highlighted the clinical potential of endolysins. Cpl-1 significantly eliminated *Streptococcus pneumoniae* from multiple serotypes, inhibited bacterial colonization in the nasopharynx, and effectively prevented acute otitis media [19,20]. Moreover, Cpl-1 could effectively rescue mice from infections caused by *S. pneumoniae* through systemic administration [21]. In comparison, endolysin SP-CHAP demonstrated more potent bactericidal activity against *S. pneumoniae* in a mouse nasopharyngeal model, demonstrating superior efficacy to Cpl-1 [22]. Several endolysins targeting multidrug-resistant *Enterococcus* have shown excellent therapeutic efficacy in mouse models. Cheng et al. found that LysEF-P10 not only increased the survival rate in mice infected with *Enterococcus faecalis*, but also restored the balance of the gut microbiota [23]. Additionally, Ply113 could significantly decrease the bacterial load in a sepsis model of mice co-infected with *E. faecium* and *S. aureus* [24]. These studies highlight the remarkable potential of endolysins in combating bacterial infections.

Endolysins hydrolyze specific sites of bacterial peptidoglycans and are generally classified into three main categories based on different catalytic mechanisms: amidases, peptidases, and glycosidases [25]. PlyC, an endolysin derived from a streptococcal bacteriophage, was composed of two separate genes, PlyCA and PlyCB. PlyCB can self-assemble into an octameric structure that is responsible for specific binding to the streptococcal cell wall. This unique structure may confer a higher lytic activity [26]. Gu et al. identified an EF-Hand-Like calcium-binding site near the groove of the active site in the catalytic domain by analyzing the crystal structure of LysGH15 [27]. The binding domain of endolysins plays a key role in bacterial cell wall attachment [28]. Zhou et al. analyzed the structure of LysIME-EF1 and revealed a unique tetrameric structure in the binding domain responsible for the translation of the truncated CBD protein via an internal ribosomal-binding site [29]. In addition, the structure of Lysostaphin SH3b demonstrated that the pentaglycine cross-bridge and peptide stem of *Staphylococcus* were recognized by independent sites of the SH3b domain [30]. These studies have elucidated the mechanism of endolysins from the perspective of structural biology. These unique endolysin structures confer distinct biological functions; thus, it is of significant importance to elucidate the structure and mechanism of action of endolysins.

In this study, we identified an endolysin, Ply2741, from an *S. suis* prophage module and evaluated its lytic activity and spectrum. The results showed that Ply2741 exhibited remarkable bactericidal activity against multidrug-resistant gram-positive bacteria. Notably, the Cys-His-Asn catalytic triad was identified as the key active region of the catalytic domain of Ply2741. Furthermore, to the best of our knowledge, the conserved residue Gln29, located near the catalytic triad, was revealed for the first time to influence the lytic activity of the endolysin. Furthermore, we elucidated the structure of the protein–substrate complex and the binding mode of Ply2741 by AlphaFold v2.0 [31], autodocking, and molecular dynamics (MD) analysis. These results demonstrate the key residues and mechanisms of the catalytic and binding domains, providing a theoretical basis for further application and modification of endolysins.

## Materials and methods

### Ethics statement

The animal experiments adhered to the ARRIVE guidelines. The protocol was approved by the Research Ethics Committee of Huazhong Agricultural University (HZAUMO-2023–0336 and HZAUMO-2024–0226). Five-week-old specific-pathogen-free (SPF) BALB/c mice were purchased from the Laboratory Animal Center of Huazhong Agricultural University (HZAU), and the experiments were conducted in the ABSL-2 laboratory at HZAU. During the experiments, mice were maintained under controlled conditions of constant temperature and humidity, with a 12-h light/dark cycle. At the end of the experiments or when mice showed signs of severe distress, euthanasia was performed via CO_2_ inhalation.

### Bacteria and cell culture conditions

All the strains used in this study are listed in Tables S1 and Table S2. *Streptococcus*, *Enterococcus*, and *Erysipelothrix rhusiopathiae* were cultured in TSB and TSA media (BD, USA) containing 10% (v/v) fetal bovine serum (Sigma-Aldrich, USA). *Salmonella, Escherichia coli,* and *Staphylococcus* were cultured in Luria-Bertani (LB) broth and LB-agar plates (Solarbio, China). A549 (ATCC: CCL-185), Huh7, and 293T (ATCC: CRL-3216) cells were cultured in Dulbecco’s modified Eagle’s medium (DMEM) (Gibco, USA) supplemented with 10% (v/v) fetal bovine serum (Sigma-Aldrich, USA) at 37°C in a 5% CO_2_ atmosphere.

### Phylogenetic analysis of endolysins

Phylogenetic analysis was performed on the amino acid sequences of 270 strains of the *Streptococcus* endolysins. The amino acid sequences of 247 endolysins were obtained from the prophage modules of *Streptococcus* species available in the NCBI database, while the remaining 23 prophage endolysin sequences are listed in Table S3. The classification criteria refer to those previously described by Oechslin et al. [32]. Briefly, open reading frames (ORFs) in *Streptococcus* prophage genomes annotated as N-acetylmuramoyl-L-alanine amidase, glycoside hydrolase, glucosaminidase, endolysin (lysin), CHAP, or lysozyme were initially identified as potential endolysins. Subsequently, the conserved domains of these potential endolysins were analyzed using SMART (http://smart.embl-heidelberg.de/). Gene fragments containing GH25, amidase, CHAP, or lysozyme in the catalytic domains were explicitly classified as endolysins [33]. A phylogenetic tree was constructed via Mega 7 using the neighbor-joining method with 1000 bootstrap replications [34]. The phylogenetic tree was visualized and modified using the Interactive Tree Of Life (https://itol.embl.de/) [35]. Additionally, the physiochemical characteristics of the endolysin were analyzed using Expasy (https://web.expasy.org/protparam/).

### Plasmid construction, protein expression, and purification

The gene encoding the endolysin of Ply2741 (GenBank accession number: PQ213358) was amplified by PCR using *S. suis* SS2741 as the template with primers specified in Table S4. Subsequently, the fragment was cloned into the pCold^TM^ II plasmid (Takara, Japan), and the recombinant plasmid pCold-Ply2741 was transformed into *E. coli* BL21 (DE3) competent cells. The recombinant strain was cultured in LB medium supplemented with 100 μg/ml ampicillin at 37°C until OD_600 nm_ = 0.6, and induced for 18 h under conditions by adding 0.6 mm Isopropyl β-D-thiogalactoside (IPTG) at 16°C. After centrifugation at 6000 ×*g* for 30 min at 4°C, the induced cells were resuspended in binding buffer (20 mm Tris, 150 mm NaCl, pH 7.5) and lysed using a cell pressure crusher (ATS ENGINEERING INC. Canada). The lysed sample was centrifuged at 10,000 ×*g* for 30 min at 4°C to remove precipitates. The supernatant was then filtered through a 0.22 μm filter, yielding an unpurified protein. For the purification step, all variant proteins were purified using affinity chromatography and size exclusion chromatography (SEC). Briefly, the recombinant protein with 6×His-tag at the N-terminal was initially purified using His-Trap FF column (Cytiva, USA) under elution buffer conditions (20 mm Tris, 150 mm NaCl, 300 mm imidazole, pH = 7.5) and further purified using a HiLoad Superdex 200 pg column (Cytiva, USA) with SEC buffer (20 mm Tris, 150 mm NaCl, pH = 7.5) following the protocols. The eluted proteins were identified using SDS-PAGE.

### Lytic spectrum and physicochemical characterization of Ply2741

The lytic spectrum of Ply2741 was determined by spot-test and turbidity reduction assay as described previously [36]. Briefly, for the spot-test assay, log-phase bacteria were mixed with TSA and TSB media, and the mixture was poured onto TSA or LB-agar plates. Subsequently, 10 μl of endolysin was spotted onto the plate, followed by overnight incubation at 37°C. For the turbidity reduction assay, different bacterial species in the log phase were resuspended in SEC buffer until the OD_600 nm_ reached 0.8–1.2, and then added to a final concentration of 25 μg/ml and 50 μg/ml of Ply2741. After incubating at 37°C for 30 min, the absorbance at OD_600 nm_ was measured to gauge the lytic activity by the reduction in OD_600 nm_.

The biological characteristics of Ply2741 were determined by a turbidity reduction assay as described previously [37]. For the optimal temperature assay, log-phase *S. suis* SS2741 cells were adjusted to OD_600 nm_ of 0.8–1.2. The Ply2741 (25 μg/ml) was added and the mixture was incubated at various temperatures for 1 h (4°C, 20°C, 37°C, 42°C, 50°C, 55°C, 60°C, 70°C, and 80°C). For the optimal pH assay, the lytic activity was tested in different buffers [20 mm sodium acetate buffer (pH 3.0 to 6.0), 20 mm sodium phosphate buffer (pH 7 to 8), and 20 mm Tris-HCl buffer (pH 9.0 to 10.0)]. Finally, the absorbance at OD_600 nm_ was measured and calculated the reduction in OD_600 nm_. All experiments were performed in triplicate. To explore the effect of EDTA on the lytic activity, Ply2741 (50 μg/ml) was pretreated with various concentration EDTA for 1 h. Then, Ply2741 was mixed with *S. suis* N15 and determined the OD_600 nm_ for 30 min at 10 min intervals. Furthermore, the effect of Ca^2+^ was also determined as describe previously [27]. Briefly, after incubation with 1 mm EDTA, the Ply2741 was dialyzed to remove excess EDTA. Subsequently, CaCl_2_ was then added to the mixture at concentrations of 0.1 mm and 1 mm. The mixture was incubated at 37°C for 30 min before measuring the reduction in OD_600 nm_. All experiments were conducted in triplicate. The calculation method for the reduction in OD_600 nm_ is as follows.

$$\mathrm{Reduction}\;\mathrm{in}\;\mathrm{OD}\;600\mathrm{nm}=\mathrm{OD}\;600\mathrm{nm}\;\;\left(\mathrm{Control}\right)-\mathrm{OD}\;600\;\mathrm{nm}\left(\mathrm{Ply}\;2741\right)$$

### Antimicrobial susceptibility testing and kinetic time kill assay

To explore the lytic activity of Ply2741 against multi-drug resistant bacteria, the MICs of *Streptococcus* (*S. suis* N15 and *Streptococcus agalactiae* ATCC13813), *Staphylococcus* (*S. aureus* S8 and *Staphylococcus epidermidis* Z17), and *Enterococcus* (*E. faecalis* 004 and 009) were tested by broth microdilution method according to the guidelines recommended by the Clinical and Laboratory Standards Institute [38]. Ampicillin, chloramphenicol, erythromycin, tetracycline, oxacillin, and vancomycin were used to test in this assay.

To evaluate the bactericidal activity of Ply2741 against different species of bacteria *in vitro*, we determined the bactericidal kinetic curve and the number of bacteria after treatment with endolysin, as described previously [39]. Briefly, the log-phase bacteria were washed three times, and Ply2741 was added (50 μg/ml). After incubation at 37°C for 30 min, samples were followed by ten-fold dilution and cultured on TSA plates for colony counting. Additionally, the bactericidal kinetic curve of Ply2741 was determined. The log-phase bacteria were resuspended in SEC buffer until the OD_600 nm_ reached 0.8 to 1.2, and then different final concentrations of Ply2741 were added. The OD_600 nm_ was determined for 1 h at 5 min intervals. All experiments were repeated three times.

### Biofilm eradication assay

The antibiofilm ability of Ply2741 against gram-positive bacteria was evaluated as described previously [40]. Briefly, log-phase bacteria were inoculated in 96-well plates and incubated at 30°C for 24 h and 48 h. The experimental groups were treated with Ply2741 at final concentrations of 50 μg/ml, followed by incubation at 37°C for 1 h and subsequent washing three times with SEC buffer. Subsequently, in some wells, biofilms were resuspended and counted on plates. For the other wells, crystal violet staining was used to evaluate the ability of Ply2741 to remove bacterial biofilms, as described previously [41]. In short, after drying at room temperature, the wells were stained by adding 200 μl crystal violet for 30 min. Finally, 33% acetic acid was added for dissolution and the absorbance at OD_590 nm_ was measured. All experiments were performed in triplicates.

### Toxicity to mammal cells and mice

To evaluate the toxicity of Ply2741, we performed experiments on cells and mice as described previously [42]. Briefly, A549, 293T, and Huh7 cells were seeded overnight at a density of 5 × 10^3^cells/well in a 96 well plate. Subsequently, Ply2741 was added to the cells at a final concentration of 200 μg/ml, and SEC buffer served as a negative control. After incubation at 37°C for 24 h, the CellTiter 96® AQueous One Solution Cell Proliferation Assay (Promega, USA) was used to detect the absorbance of OD_490 nm_ according to the protocol, and all experiments were repeated three times.

Mice were used to evaluate the safety of Ply2741 *in vivo*. Five-week-old specific-pathogen-free (SPF) BALB/c mice were randomly divided into three groups (*n* = 3). Subsequently, the mice were injected with 500 μg of Ply2741, with a phosphate buffered saline (PBS) buffer serving as control. The mice in the mock group did not receive any treatment. The physiological status and survival rate of mice were monitored daily. After 7 d, the tissues were collected for histopathological analysis.

### Transmission electron microscopy (TEM)

To investigate the bacterial morphology after treatment with Ply2741, we observed it by TEM. Briefly, log-phase *S. suis* SC19 cells were washed and treated with Ply2741. Subsequently, cells were cultured at 37°C for 5 min, 15 min, and 30 min. Untreated bacteria served as controls. Subsequently, the samples were fixed with 2.5% glutaraldehyde, dehydrated in ethanol, and embedded in SPI-Pon 812 resin for cutting using UC6 ultra-microtome (Leica Microsystems, Austria). Observations were conducted using an HT7800 transmission electron microscope (Hitachi, Tokyo, Japan).

### Scanning electron microscopy (SEM)

To explore the morphology of bacteria biofilms treated with Ply2741, we performed SEM experiments. Briefly, bacteria were inoculated into a 24-well plate with cell coverslips at the bottom and cultured at 30°C for 48 h. After washing with SEC buffer, Ply2741 at final concentration of 25 μg/ml was added to the experimental groups, and SEC buffer served as a control. After washing with SEC buffer to remove planktonic bacteria, 2.5% glutaraldehyde was added for 30 min. Finally, the coverslips were sprayed with gold and observed by SEM (NTC JSM-6390LV, Japan).

### Mouse Streptococcus suis infection model

In a survival experiment, 5-week-old female SPF BALB/c mice were randomly divided into five groups (*n* = 9 per group) and each group was intraperitoneally challenged with 1 × 10^9^ CFU of *S. suis* SC19. After 1-h post-infection, mice were treated via intraperitoneal injection with various dose of Ply2741 (100 μg, 200 μg, and 400 μg), ampicillin (5 mg/kg), or PBS, respectively. The survival rate of the mice was monitored for 7 d.

Additionally, the therapeutic efficacy of Ply2741 administered via various injection routes after 3 h challenge was further evaluated. Mice were randomly divided into four groups: control group (*n* = 9), SC19 group (*n* = 9), intraperitoneal injection group (i.p.) (*n* = 9), and intramuscular injection group (i.m.) (*n* = 9). Each group was inoculated with 5 × 10^8^ CFU of SC19, while the control group received PBS buffer. At 3 h post-infection, mice were treated with 200 μg of Ply2741 via intraperitoneal or intramuscular injection. Mouse organs were collected at 6 h and 5 d post-treatment and homogenized in a PBS buffer to determine the bacterial load by ten-fold serial dilution on TSA plates. Inflammatory mediator levels (*TNF-α*, *IL-6*, *IL-1β*, and *IFN-γ*) were measured in mouse serum at 6 h post-treatment using an ELISA kit (Wuhan ColorfulGene Biological Technology Co., Ltd., China).

### Structure prediction and analysis of Ply2741 domains

To explore the mechanism of action of Ply2741, the three-dimensional structures of CD and CBD were analyzed. Swiss-model was used to identify similar endolysin structures (https://swissmodel.expasy.org/) [43]. The amino acid sequences exhibiting high similarity to CD and CBD were subjected to multi-sequence alignment using ClustalW v2.1 (https://www.ebi.ac.uk/Tools/msa/clustalo/). The alignment results were modified using ESPript v3.0 (https://espript.ibcp.fr/ESPript/cgi-bin/ESPript.cgi) [44]. The three-dimensional structures of the wild-type and mutant protein were predicted by artificial intelligence using the Colab server (https://colab.research.google.com/github/sokrypton/ColabFold/blob/main/AlphaFold2.ipynb#scrollTo=11l8k–10q0C) [45], a simplified version of AlphaFold v2.0. The reliability of all prediction results was evaluated using internally predicted Local Distance Difference Test score (pLDDT). ConSurf (https://consurf.tau.ac.il/consurf_index.php) was used to analyze the conserved surface regions. The PDBsum Generate was used to generate the topology and Ramachandran plot (PDBsum Generate (ebi.ac.uk)). All protein structures were analyzed using PyMol (PyMOL Molecular Graphics System, Schrodinger LLC).

### Site-directed mutagenesis and bactericidal activity of mutant endolysin

To further explore the role of the key residues, site-directed mutagenesis was conducted, and the bacterial activity of the mutant protein was determined. Briefly, the residues were modified to alanine using the Mut Express II Fast Mutagenesis Kit (Vazyme, China) according to the manufacturer’s protocol. The primers used are listed in Table S4. The expression and purification protocols for the protein mutants were identical to those of the wild-type.

### Circular dichroism analysis

To analyze the secondary structure after key residue mutations in the catalytic domain, circular dichroism analysis was carried out using a JASCO J-1500 spectropolarimeter (JASCO, Japan). In brief, the wild-type and mutant proteins were adjusted to 0.5 mg/ml with SEC buffer, and 0.1 cm quartz cup was used to determine at 200–260 nm absorbance.

### Nano differential scanning fluorimetry (nanoDSF)

To explore the difference in thermal stability after the mutation of key residues in the catalytic domain, nanoDSF experiments were carried out using Prometheus NT.48 (Nano Temper, Germany). Briefly, after adjusting the wild type and mutant proteins to 0.5 mg/ml using SEC buffer, Standard Capillary Chips were used to load samples (Nano Temper, Germany). The temperature was increased from 20°C to 95°C at a rate of 1°C/min.

### Molecular docking and molecular dynamics analysis

Based on the molecular structure of peptidoglycan, the candidate substrate molecules, L-alanyl-D-isoglutamine (PubChem CID: 7019962), P4 (L-Ala-D-iGln-L-Lys-D-Ala), P4-2A (L-Ala-D-iGln-L-Lys-(L-Ala-L-Ala)-D-Ala), and P4-G5 (L-Ala-D-iGln-L-Lys-(Gly-Gly-Gly-Gly-Gly)-D-Ala) were identified [46]. To analyze the binding affinities and modes, we conducted molecular docking as described previously [47]. Briefly, molecular structures were obtained from the PubChem database (https://pubchem.ncbi.nlm.nih.gov/) or drawn using ChemDraw (Cambridge Soft, USA). Subsequently, the structures were converted into PDBQT format. Hydrogen atoms were added after removing all the water molecules. The grid box covers each protein domain, with the docking pocket set as a square pocket of 30 Å × 30 Å × 30 Å and a grid spacing of 0.05 nm. Molecular docking was performed using Autodock Vina 1.2.2 (https://autodock.scripps.edu/). Discovery Studio (Dassault Group, France) was used to analyze the complex structure. Additionally, MD analysis was performed to determine the binding reliability of the substrate molecule with the highest docking score using the GROMACS 5.1.4 program package [48]. The topological structure of Ply2741CBD as generated using the Amber 99Sb-ILDN force field and TIP3P water model [49], and the ligand topological structures were prepared using the ACPYPE server [50]. For each MD analysis system, a simulation was run for 100 ns at 310 K and 1 bar atmospheric pressure. Finally, the root-mean-square fluctuation (RMSF) and root-mean-square deviation (RMSD) were analyzed using GROMACS tools.

### GFP-tagged Ply2741CBD wild type and mutant plasmid construction, expression, and purification

Briefly, the Ply2741CBD fragment was amplified by PCR using *S. suis* SS2741 as the template and the eGFP fragment was cloned by PCR using the pCAG-GFP plasmid (Addgene: #11150) as the template. The Ply2741CBD-eGFP fragment was further amplified by overlap PCR using Ply2741CBD and eGFP fragments as templates, and then inserted into the pCold^TM^ II expression plasmid. Site-directed mutagenesis recombinant plasmids were constructed according to the protocols described above.

### Fluorescence-activated cell sorting (FACS) analysis

To explore the binding ability of Ply2741CBD after key residue mutations in the CBD, FACS experiments were performed [29]. Logarithmic-phase *S. aureus* S8 was adjusted to 10^7^CFU/ml and washed thrice with SEC buffer. The bacteria were resuspended in eGFP-tagged Ply2741CBD wild-type and mutant proteins, and the SEC buffer served as a control. After the incubation at 37°C for 15 min, the samples were centrifugated at 5000 × g for 2 min and washed six times before measurement. The samples were analyzed using Cytoflex-LX according to the manufacturer’s protocol (Beckman Coulter, USA).

## Statistical analysis

Data are expressed as mean ± standard deviation (SD). Data were tested for normality using the Anderson–Darling test (*n* > 6) and Quantile–Quantile plots (*n* < 6). Statistical analysis and comparison were performed using the unpaired two-tailed Student’s t-test, one- and two-way analysis of variance (ANOVA) via GraphPad Prism 8 (GraphPad Software, USA). Significant differences in survival experiment were analyzed by log-rank (Mantel-Cox) test. The number of experimental replicates is presented in the figure legends. *p values <* 0.05 (*) were considered statistically significant.

## Results

### The diversity of endolysins in Streptococcus prophages genome

**Figure 1.**
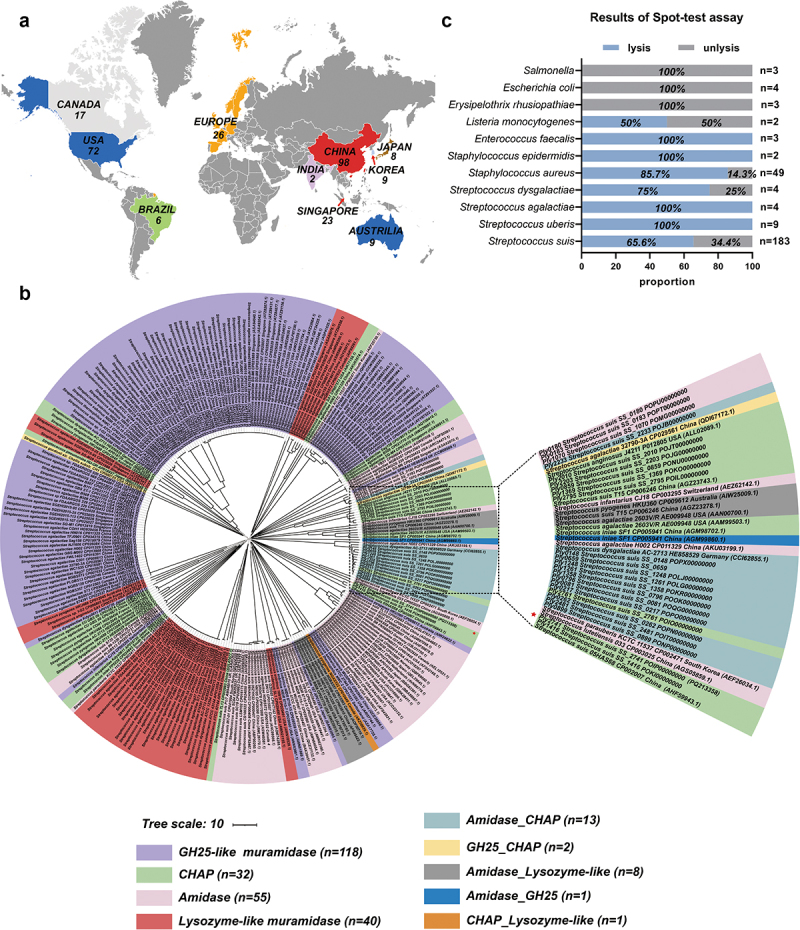
Phylogenetic relationships of endolysins from the *Streptococcus* genus and antibacterial activity of Ply2741 against different genus bacteria. (a) Global distribution of endolysins from the *Streptococcus* genus involved in phylogenetic relationships analysis. Different colors correspond to various countries and regions and the quantities of endolysins isolated from each country are indicated in the figure. Figures were generated using Adobe illustrator (Adobe Inc., USA). (b) Phylogenetic tree of S*treptococcus* endolysins. The phylogenetic tree was generated using Mega 7 and visualized using iTOL. Conserved structural domains was determined based on SMART predictions. The endolysins were classified into nine types based on catalytic domains and represented by different colors, with protein IDs labeled in the figure. Ply2741, located on a separate branch, was marked with a red asterisk. (c) Spot-test activity of Ply2741 against various bacteria. Purified Ply2741 was spotted onto bacterial lawns and incubated at 37°C for 12 h. The presence or absence of inhibition zones was observed, and the proportion was calculated accordingly. The details of results were shown in Table S1.

To analyze the diversity of phage endolysins within the *Streptococcus* genus, we conducted a phylogenetic analysis of their amino acid sequences. These endolysins exhibited a broad geographical distribution (Figure 1a). Phylogenetic tree and conserved domain analysis results revealed that the catalytic domains (CDs) of endolysins could be classified into nine distinct types (Figure 1b). These CDs are composed of GH25, CHAP, amidase, and lysozyme-like muramidase in a random manner. Furthermore, the phylogenetic analysis results suggested that there was no consistent pattern in the domain composition of endolysins among different strains of *Streptococcus*, indicating no correlation between bacterial species and the structural composition of the evaluated endolysins. These findings highlight the extensive diversity of prophage endolysins in the *Streptococcus* spp. Additionally, in terms of evolutionary relationships, Ply2741 was located on a distinct branch, indicating that it is a new endolysin (Figure 1b). These findings prompted us to conduct an in-depth study of Ply2741.

### Biological characterization and lytic spectrum of Ply2741

A prophage endolysin, Ply2741, has been identified in *S. suis* SS2741. The results showed that the theoretical PI, instability index, and GRAVY of Ply2741 were 9.25, 26.96, and −0.344, respectively, implying that Ply2741 was soluble and stable (Table S5). Conserved domain analysis revealed that Ply2741 is composed of cysteine- and histidine-dependent amidohydrolase/peptidase (CHAP) CD and SH3b CBD (Figure S1a). After size exclusion chromatography purification (Figure S1b), the biological properties of Ply2741 were analyzed, and the activity was maintained at temperatures of 4–50°C and pH levels of 5–8 (Figure S1c and S1d). Additionally, as shown in Figure S1e, EDTA (>0.01 mm) could significantly reduce the lytic activity of Ply2741. Surprisingly, the activity of Ply2741 was fully restored after the re-addition of 0.1 mm CaCl_2_, indicating that the lytic activity of Ply2741 is dependent on the presence of calcium ions (Figure S1f).

The lytic spectrum of Ply2741 was assessed qualitatively using a spot-test and quantitatively using turbidity reduction assays. The spot-test results demonstrated that Ply2741 exhibited clear inhibitory halos against gram-positive bacteria, including *S. suis* (65.6%, *n* = 183), *S. aureus* (85.7%, *n* = 49), and *E. faecalis* (100%, *n* = 3) (Figure 1c and Table S1). Additionally, as depicted in Figure S1G, for *Streptococcus*, different serotypes of *S. suis* were sensitive to Ply2741, exhibiting a reduction in OD_600 nm_ ranging from approximately 0.4 to 0.9 after exposure to Ply2741 at a concentration of 50 µg/mL for 30 min. Other streptococci, including *S. agalactiae*, *S. pneumoniae*, and *Streptococcus uberis*, also showed a reduction in OD_600 nm_ to values greater than 0.7 under similar conditions. However, the activity of Ply2741 against *Streptococcus dysgalactiae* was comparatively lower, with a reduction in OD_600 nm_ to less than 0.6. With *Staphylococcus*, Ply2741 could lysed all tested strains, resulting in a reduction in OD_600 nm_ to 0.7. Ply2741 also exhibited lytic against *E. faecalis*, the reduction in OD_600 nm_ was reached to 0.8. However, no significant effect was observed when Ply2741 was applied to gram-negative bacteria. Collectively, these findings highlight the broad lytic spectrum of Ply2741.

### Ply2741 exhibited lytic activity against multidrug-resistant gram-positive bacteria in vitro

**Figure 2.**
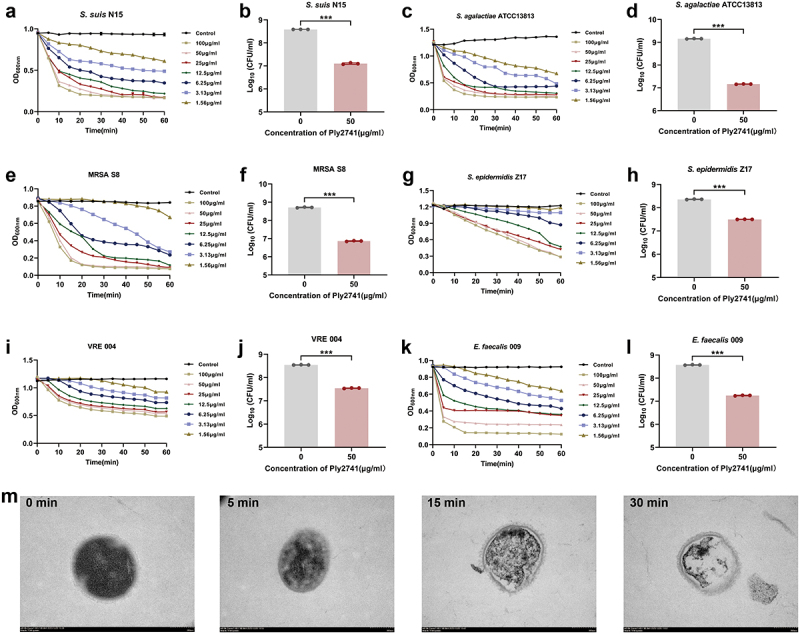
Bactericidal activity of Ply2741 against multidrug-resistant gram-positive bacteria *in vitro*. (a, c, e, g, i, k). Bactericidal kinetic curves of Ply2741 against *S. suis* N15 (a), *S. agalactiae* ATCC13813 (c), MRSA S8 (e), *S. epidermidis* Z17 (g), VRE 004 (i), and *E. faecalis* 009 (k) at different concentrations within 60 min. (b, d, f, h, j, l). Bacterial counts after treatment with Ply2741 (50 μg/ml) for 30 min against multidrug-resistant strains, with SEC buffer as control. All experiments are expressed as the mean ± SD of three independent replicates. Statistical analysis was performed using Student’s t-test, with different asterisks representing levels of significance. *, *p* < 0.05, **, *p* < 0.01, and ***, *p* < 0.001. (m). Transmission electron microscopy observation of the morphological changes of *S. suis* SC19 after treatment with Ply2741 at different time points, with images magnified at × 40000.

As shown in Table S6, *S. suis N15*, *S. aureus S8* (MRSA), *S. epidermidis* Z17, *E. faecalis* 004 (VRE), and *E. faecalis* 009 were multidrug-resistant bacteria. To evaluate the lytic efficiency of Ply2741 against multidrug-resistant bacteria, we determined its bactericidal activity against different genera of bacteria at various concentrations and incubation times. Ply2741 rapidly reduced the OD_600 nm_ within 15 min against *Streptococcus* (including multidrug-resistant *S. suis* N15 and *S. agalactiae* ATCC13813) (Figure 2a,c). Specifically, incubation with Ply2741 at a concentration of 50 μg/ml for 30 min significantly reduced the bacterial count, with *S. suis* N15 decreased from 8.59 log_10_CFU/ml to 7.10 log_10_CFU/ml (*p* < 0.001), and *S. agalactiae* ATCC13813 decreased from 9.15 log_10_CFU/ml to 7.17 log_10_CFU/ml (*p* < 0.001) (Figure 2b,d). For *Staphylococcus*, Ply2741 effectively eliminated MRSA S8 within 15 min, and *S. epidermidis* Z17 within 60 min (Figure 2e,f). Additionally, 50 μg/ml of Ply2741 caused a significant reduction in count of MRSA S8 (*p* < 0.001) and *S. epidermidis* Z17 (*p* < 0.001) (Figure 2f,h). Notably, Ply2741 significantly eradicated VRE 004 and *E. faecalis* 009 within 20 min (Figure 2i,k). Furthermore, the incubation with Ply2741 led to a significant reduction in bacterial counts: for VRE 004, the count decreased from 8.54 log_10_CFU/ml to 7.54 log_10_CFU/ml (*p* < 0.001), and for *E. faecalis* 009, the count decreased from 8.57 log_10_CFU/ml to 7.25 log_10_CFU/ml (*p* < 0.001) (Figure 2j,l). The morphological changes in *S. suis* exposed to Ply2741 were analyzed by TEM, revealing the complete release of internal contents and formation of a classic “ghost” after 30 min of incubation (Figure 2m). In conclusion, these results indicate that Ply2741 exhibited significant dose and time-dependent bactericidal activity against multidrug-resistant gram-positive bacteria.

### Eradication effects of Ply2741 against biofilms

**Figure 3.**
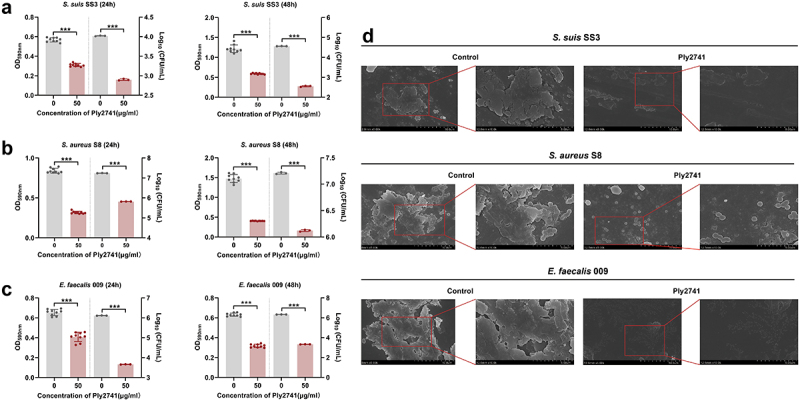
Effect of Ply2741 on bacterial biofilms. (a, b, c) Absorbance at OD_590 nm_ and viable cells numbers of 24 h and 48 h-biofilms of *S. suis* SS3 (a), MRSA S8 (b), and *E. faecalis* 009 (c) after treatment with Ply2741 at 50 μg/ml for 1 h, with SEC buffer as control. All experiments are expressed as the mean ± SD of three independent replicates. Statistical analysis was performed using Student’s t-test, with different asterisks representing levels of significance. *, *p* < 0.05, **, *p* < 0.01, and ***, *p* < 0.001. (d) SEM images of 48 h-biofilms of different bacteria after treatment with Ply2741 (at a 25 μg/ml final concentration), magnified at × 5000 (left image) and x10000 (right image).

To explore the antibiofilm ability of Ply2741, bacterial counting, crystal violet staining method, and SEM experiments were carried out. Crystal violet experiments at 24 h and 48 h biofilms showed a significant reduction in absorbance at OD_590 nm_ after Ply2741 treatment (*p* < 0.001) (Figure 3a–c). The bacterial count results revealed a notable decrease in viable bacteria of biofilms after Ply2741 treatment compared to the control. Specifically, for *S. suis* SS3, the counts decreased from 4.02 log_10_CFU/ml to 2.90 log_10_CFU/ml at 24 h and from 4.56 log_10_CFU/ml to 2.55 log_10_CFU/ml at 48 h (Figure 3a). Similarly, for *S. aureus* S8, counts were reduced from 7.24 log_10_CFU/ml to 5.82 log_10_CFU/ml at 24 h and from 7.21 log_10_CFU/ml to 6.12 log_10_CFU/ml at 48 h (Figure 3b). For *E. faecalis* 009, reductions were from 6.11 log_10_CFU/ml to 3.66 log_10_CFU/ml at 24 h and from 6.33 log_10_CFU/ml to 3.33 log_10_CFU/ml at 48 h (Figure 3c). In addition, SEM images further demonstrated that Ply2741 significantly eradicated established biofilms (Figure 3d). Collectively, these results indicate that Ply2741 exhibits a significant ability to eradicate biofilms *in vitro*.

### Therapeutic effect of Ply2741 in a mouse model infected with *S. suis*

**Figure 4.**
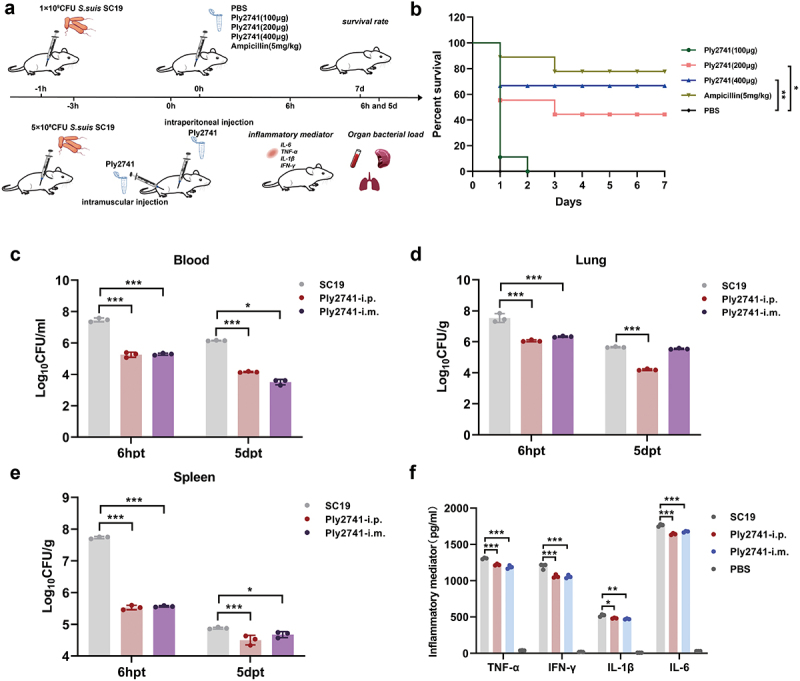
Significant therapeutic efficacy of Ply2741 in a mouse model of *S. suis* SC19 infection *in vivo*. (a) Schematic diagram of the experimental procedure. (b) Survival rate of SPF BALB/c mice infected with 1 × 10^9^CFU of SC19 and treated with different dose of Ply2741 at 1 h post-infection, ampicillin and PBS served as the positive and negative control, respectively. The survival rate was observed for 7 d. (c, d, e) Bacterial load in blood (c), lungs (d), and spleens (e) of mice treated with 200 μg of Ply2741 via different administration routes after 3 hours of low-dose SC19 (5 × 10^8^CFU) infection. (f) Levels of inflammatory mediators *tnf-α*, *IL-1β, IL-6*, and *ifn-γ* in mouse serum after intraperitoneal and intramuscular treatment of Ply2741. Data were expressed as mean ± SD. Statistical analysis was performed using log-rank (Mantel-Cox) test and two-way ANOVA followed by Tukey’s multiple comparison test, with different asterisks representing levels of significance. *, *p* < 0.05, **, *p* < 0.01, and ***, *p* < 0.001.

We further explored the therapeutic effects of Ply2741 against *S. suis* SC19 infection *in vivo* (Figure 4a). Prior to the experiment, the safety of Ply2741 was evaluated in both mammalian cells and mice. No significant damage was observed in either the cells (Figure S2a) or mouse tissues (Figure S2b), confirming the safety of Ply2741.

In the survival experiment, all mice challenged with 1 × 10^9^CFU of *S. suis* SC19 succumbed within 12 h. However, treatment with Ply2741 significantly improved survival rates in a dose-dependent manner (Figure 4b). Ply2741 at doses of 200 μg and 400 μg provided survival rates of 44% (*p* < 0.05) and 67% (*p* < 0.01), respectively, while ampicillin exhibited a survival rate of 78% (Ply2741 (400 μg) vs. ampicillin, *p* > 0.05). Similarly, experiments with different treatment routes at 3 h post-challenge (5 × 10^8^CFU of SC19) showed significant reductions in bacterial load. As shown in the Figure 4c, after 6 h and 5 d of treatment, the bacterial load in the blood of mice treated via intraperitoneal injection decreased by 2.21 log_10_CFU/ml and 2.17 log_10_CFU/ml, respectively, while intramuscular injection resulted in decreases of 2.00 log_10_CFU/ml and 2.66 log_10_CFU/ml. Comparable therapeutic effects were observed in the lungs and spleen (Figure 4d,e). Furthermore, the inflammatory mediator levels of *TNF-α* (i.p.: *p* < 0.001; i.m.: *p* < 0.001), *IL-6* (i.p.: *p* < 0.001; i.m.: *p* < 0.001), *IL-1β* (i.p.: *p* < 0.05; i.m.: *p* < 0.01), and *IFN-γ (i.p.: p < 0.001; i.m.: p < 0.001)* were also decreased significantly (Figure 4f). In conclusion, these results demonstrate that Ply2741 exhibits a significant therapeutic effect against *S. suis* infection *in vivo*.

### The catalytic activity of Ply2741 depends on the classical “Cys-His-Asn” catalytic triad

**Figure 5.**
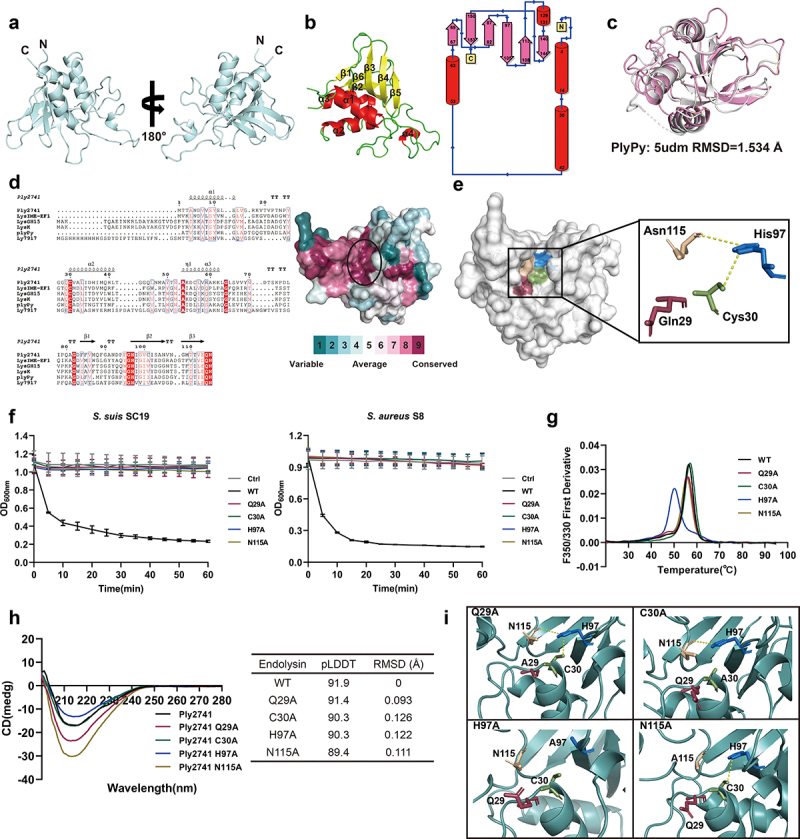
Structural and functional analysis of the catalytic domain of Ply2741. (a) The overall structure of Ply2741CD depicted in cartoon model. (b) Secondary and topology structure of Ply2741CD. α-helices, β-strands, and coil are indicated in red, yellow, and green, respectively. (c) The catalytic domain structural comparison between Ply2741 (white) and PlyPy (pink) (PDB id:5udm) using PyMOL, with an RMSD of 1.543Å. (d) Multiple sequence alignment of Ply2741CD with homologous proteins and conserved surface analysis of Ply2741CD. The alignment was performed using ClustalW and visualized using ESPript. Strictly conserved residues are highlighted in red, with the protein secondary structure shown above the sequence. (e) Surface representation of Ply2741CD, with key catalytic residues displayed in stick model and labeled with different colors. A zoomed view of “Cys-His-Asn” catalytic triad and Gln29. (f) Bactericidal curves of wild type and catalytic residues mutant proteins against *S. suis* SC19 and MRSA S8. SEC buffer was used as a control. Experimental data are presented as mean ± SD and were independently repeated three times. (g) Thermal stability of wild type and mutant endolysins, with proteins at a concentration of 0.5 mg/ml determined for melting temperatures. The experiment was independently repeated three times, and Figure 5g represents data from one of the three independent experiments. (h). Structural analysis of wild-type Ply2741CD and its mutant proteins. Secondary structure analysis of endolysins were determined via circular dichroism at 200-260 nm. The protein structures predicted by AlphaFold were compared using PyMOL, with RMSD indicating the differences between the proteins. (i) Interaction of key catalytic residues in catalytic active sites in wild-type and mutant Ply2741CD, with key residues displayed in stick model.

To further explore the lytic mechanism of Ply2741, we analyzed the structure of Ply2741CD using ColabFold. The optimal CD structure is shown in Figure 5a, with a pLDDT index of 91.9 (Figure S3a). The Ramachandran plot results showed that 90.2% of residues were located in the most favored regions, with 9.8% in the allowed regions (Figure S3b), indicating accurate modeling of Ply2741CD. In addition, the secondary structure and topological analysis revealed four α-helices and six β-strands in Ply2741CD, with two loop structures between α1 and α2, and between β4 and β5 (Figure 5b). These loops were positioned externally to the core region and oriented on the same side of the overall structure. Structural comparisons with PlyPy (PDB ID: 5udm) revealed similarities (RMSD = 1.534Å), with differences primarily in the flexible loop regions (Figure 5c).

Multiple sequence alignment and structural analysis of conserved surfaces revealed a narrow and deep groove formed by conserved residues within Ply2741CD, suggesting that this region may be associated with catalytic activity (Figure 5d). Further investigation identified a classical catalytic triad consisting of Cys30, His97, and Asn115 within this conserved groove, which are likely catalytic active sites of Ply2741. Additionally, the nearby residue Gln29 is also highly conserved and contributes to the formation of the conserved groove along with the catalytic triad (Figure 5e). To validate the function of these residues, site-directed mutagenesis was performed by mutating Gln29, Cys30, His97, and Asn115 to Alanine, which was identified by SDS-PAGE (Figure S4a and S4b). The lytic activity results indicated that Ply2741 Q29A, C30A, H97A, and N115A were almost completely inactive compared to Ply2741 wild-type (Ply2741WT) (Figure 5f). These results indicate that, in addition to the catalytic triad residues, the conserved residue Gln29 near the catalytic active site also contributes to the bactericidal activity of Ply2741.

Furthermore, nanoDSF results showed that the mutation of His97 within the catalytic triad significantly reduced the thermal stability of Ply2741, decreasing the melting temperature by 6.4°C (Figure 5g). To further investigate the mechanism of active site mutations on protein stability, structural analysis of wild-type and mutant Ply2741CD proteins was performed. Circular dichroism results revealed that there were no significant differences between the wild-type and mutant proteins, with nearly identical overall curves (Figure 5h). The structures of the mutant proteins, as predicted using AlphaFold, were compared to the wild-type and showed no significant differences in overall structure, with a maximum RMSD of 0.126Å, indicating that mutations in the catalytic active sites do not affect the overall structure (Figure 5h). However, structural comparisons of the active center region revealed that the mutation of His97 disrupted hydrogen bonding with Cys30 and Asn115 (Figure 5i), suggesting that the loss of interactions among the catalytic triad residues significantly decreases the overall stability and lytic activity of the endolysin.

### Binding mechanism of Ply2741CBD to substrate molecules

**Figure 6.**
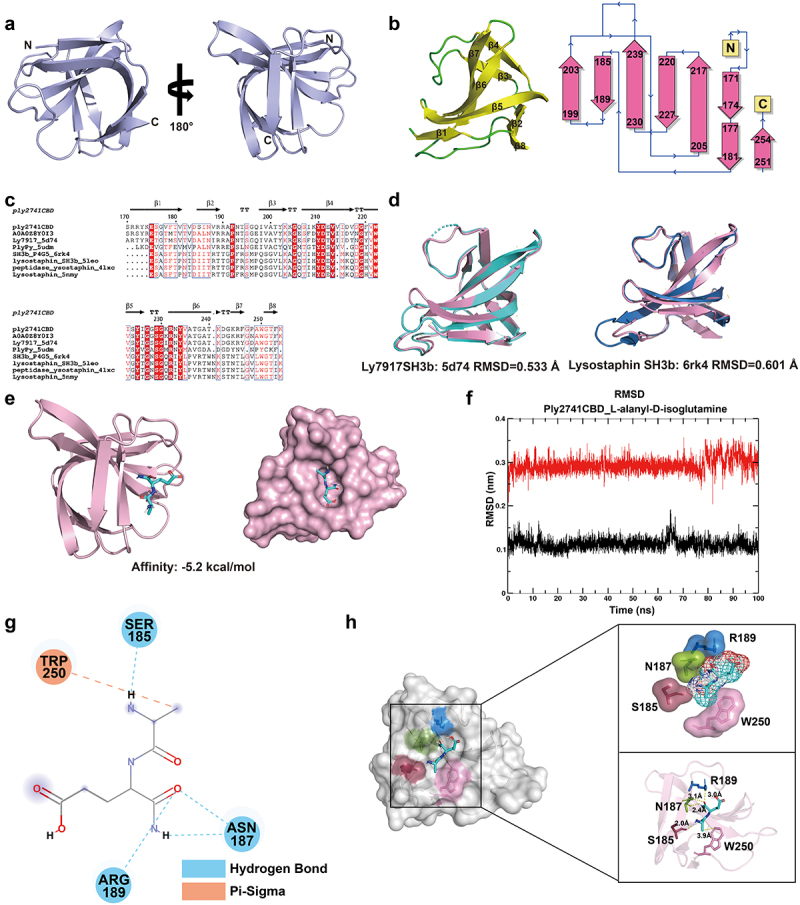
Structural analysis of the Ply2741CBD and the substrate complex. (a) The overall structure of Ply2741CBD depicted in cartoon model. (b) Secondary and topology structure of Ply2741CBD. β-strands and coil are indicated in yellow and green, respectively. (c) Multiple sequence alignment of Ply2741CBD with homologous proteins. (d) Structural comparison between Ply2741CBD (pink), Ly7917 (cyan) (PDB ID:5d74), and Lysostaphin SH3b (blue) (PDB ID: 6rk4) with an RMSD of 0.533Å and 0.607Å, respectively. (e) Structure of the Ply2741CBD_L-alanyl-D-isoglutamine complex shown in both cartoon and surface model, obtained through molecular docking. (f) The RMSD results of the Ply2741CBD_L-alanyl-D-isoglutamine complex structure over 100 ns obtained from molecular dynamics analysis. The protein and ligand were represented by black and red curves, respectively. (g) Key residues involved in the interaction between Ply2741CBD and L-alanyl-D-isoglutamine. S185, N187, and R189 interact with ligand through hydrogen bonds (blue), while W250 interacts with ligand through a π–sigma interaction (orange). (h) Surface representation of the Ply2741CBD_L-alanyl-D-isoglutamine complex, with key residues displayed in stick model and labeled with different colors. The zoomed image illustrates the interaction between L-alanyl-D-isoglutamine and key residues of CBD, depicted in both dots and stick model. Distances between residues and L-alanyl-D-isoglutamine were measured via PyMOL and labeled in the image.

The highest-ranked CBD structure is presented in Figure 6a, with a pLDDT score of 97.9 (Figure S3c). The Ramachandran plot results showed that 92.9% of the amino acids were located in the most favored regions (Figure S3d), indicating exceptional accuracy in modeling the Ply2741CBD structure. Additionally, secondary structure and topological analysis showed that the CBD consisted of eight β-strands arranged in an antiparallel fashion (Figure 6b). Interestingly, Ply2741CBD showed significant similarity with Ly7917 SH3b (161-245aa) (PDB ID: 5d74) and Lysostaphin SH3b (402-493aa) (PDB ID: 6rk4) in both sequence (73.49% and 39.51% similarity) and structure (RMSD = 0.533Å and 0.607Å, respectively) (Figure 6c,d), suggesting that Ply2741CBD belongs to the classical SH3b domain and may share a similar binding mechanism.

Consequently, we sought to explore the mechanism underlying CBD-peptidoglycan (PG) interactions. The candidate substrate molecules (L-alanyl-D-isoglutamine, P4, P4-2A, and P4-G5) were identified based on the structural composition of peptidoglycan (Figure S5a). Subsequently, molecular docking was performed between Ply2741CBD and the candidate substrates using AutoDock Vina. The binding affinities of Ply2741CBD docked with each substrate are shown in Table S7, and the models with the highest binding affinities are further analyzed. As shown in Figure 6e and S5b, all substrate molecules were predominantly positioned in the pocket of the CBD, suggesting that this pocket may be the main region for CBD to bind with peptidoglycan.

Given the dynamic characteristics of protein-ligand binding, we further analyzed the complexes using molecular dynamics analysis. The RMSD and RMSF of Ply2741CBD and ligands in the complexes were determined by 100 ns MD analysis via GROMACS. The RMSD trajectory serves as a valuable metric for evaluating the dynamics and stability of both the proteins and ligands in the complex. The results illustrated that in the Ply2741CBD_L-alanyl-D-isoglutamine complex structure, both the protein and ligand maintained a highly stable state. Specifically, the L-alanyl-D-isoglutamine ligands exhibited minimal fluctuations, approximately 0.05 nm, from the 75 ns to 100 ns (Figure 6f). Conversely, the stability of ligands in the Ply2741CBD_P4, Ply2741CBD_P4-2A, and Ply2741_P4-G5 structure showed a significant decrease compared to Ply2741CBD_L-alanyl-D-isoglutamine, with fluctuations observed within the range of 0.1 nm to 0.3 nm over 100 ns (Figure S6a–S6c). Additionally, the RMSF results revealed similar RMSF fluctuations in all complexes, yet each residue in the Ply2741CBD_L-alanyl-D-isoglutamine complex exhibited reduced fluctuations (Figure S6d). These findings indicate that the Ply2741CBD_L-alanyl-D-isoglutamine complex was notably more stable and deemed a more suitable substrate-binding molecule for Ply2741CBD.

### The key residues in Ply2741CBD that interact with L-alanyl-D-isoglutamine

**Figure 7.**
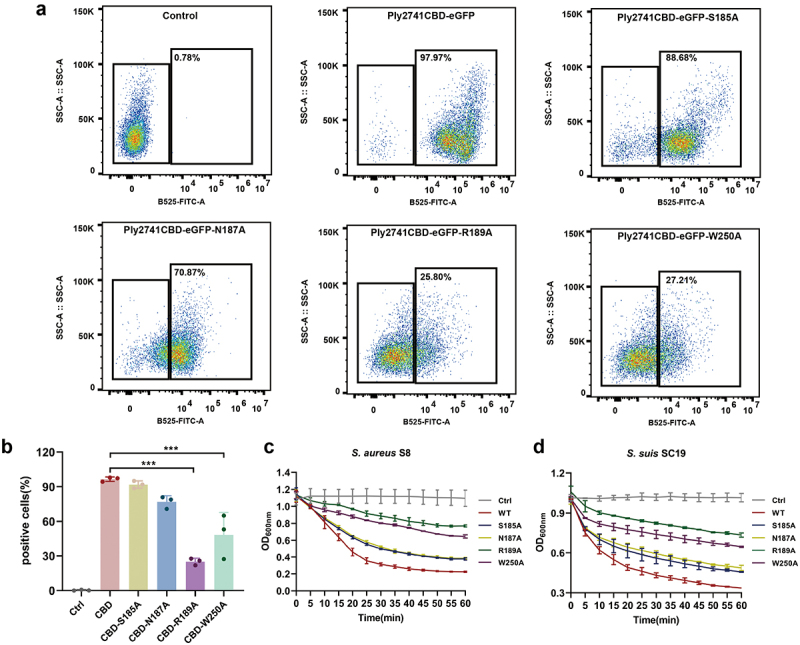
Key residues of Ply2741CBD involved in binding to bacteria. (a, b) FACS results of wild-type and binding residues mutant cbd-eGFP proteins binding to MRSA S8. Figure 7a was obtained from one of the three independent experiments. (c, d) Bactericidal curves of wild-type and binding residues mutant proteins against *S. suis* SC19 (c) and MRSA S8 (d) Data are presented as mean ± SD. Statistical analysis was performed using one-way ANOVA followed by Tukey’s multiple comparison test, with different asterisks representing levels of significance. *, *p* < 0.05, **, *p* < 0.01, and ***, *p* < 0.001.

To identify the key residues involved in the interaction between Ply2741CBD and L-alanyl-D-isoglutamine, the structure of Ply2741CBD_L-alanyl-D-isoglutamine complex was analyzed using Discovery Studio. The results revealed that L-alanyl-D-isoglutamine formed hydrogen bond interactions and π–sigma interactions with key residues (Figure 6g). Specifically, the residues Ser185, Asn187, and Arg189 exhibited hydrogen-bonding interactions, whereas Trp250 displayed π–sigma interactions with the substrate, all within a distance of less than 4 Å (Figure 6h). These interactions are crucial for maintaining complex stability and function. To determine the function of the key residues, the eGFP fragment was fused to Ply2741CBD, and site-directed mutagenesis was performed (Figure S4c-S4e). After purifying wild-type Ply2741CBD-eGFP and mutant proteins, the binding activity of CBD to *S. aureus* S8 was evaluated by fluorescence-activated cell sorting (FACS). Mutations S185A and N187A exhibited a slight impact on the binding ability, whereas R189A and W250A significantly diminished the binding ability of CBD (Figure 7a,b). In addition, the CBD mutation was transferred to a wild-type Ply2741, and the activity of mature protein after binding residue mutation was assessed. The results demonstrated a significant decrease in lytic activity against *S. suis* SC19 and *S. aureus* S8 for mutants R189A and W250A compared to the wild-type (Figure 7c,d), which may be attributed to the loss of binding ability. However, the activity of the variant protein was not completely lost, even though R189A and W250A led to the most important efforts. These results were consistent with those of the FACS experiment, indicating that Arg189 and Trp250 play a key role in Ply2741 binding to the bacterial cell wall, but no single mutation completely abolished lytic activity.

## Discussion

Phage endolysins, which are emerging as novel antimicrobial agents, have attracted considerable attention in the scientific community [51]. The modular domain composition of the endolysins can vary [52]. In this study, phylogenetic analysis was conducted on *Streptococcus* prophage-derived endolysins, revealing a correlation between evolutionary relationships and structural domain composition. The CDs were recombined and fused to produce nine types of endolysins, indicating a wide range of compositional diversity. Furthermore, our findings suggest that the structural domains of endolysins are not strongly associated with specific bacterial genera. The diversity of endolysins may arise from gene exchanges during bacterial-phage interactions, resulting in distinct endolysin domains in the bacterial genome. Similar results were reported in a study by Oechslin et al. on endolysins derived from *Lactobacillu*s [32].

Phylogenetic tree analysis showed that the endolysin Ply2741 was located on a separate branch and exhibited inhibition of halos against *Streptococcus*, *Staphylococcus*, and *E. faecalis*. Notably, bactericidal kinetic curves revealed a rapid and dose-dependent reduction in bacterial turbidity, underscoring its broad-spectrum and efficient lytic activity. Endolysins Ly7917 [53], ply1228 [54], and ply5218 [55], derived from *S. suis* prophage, exhibited lytic activity against *Streptococcus* but showed no lytic effects on *Staphylococcus* and *Enterococcus*. PlySs2 exhibits an exceptionally broad spectrum of bactericidal activity, making it the most versatile lysin reported to date [56]. In contrast, Ply2741 shows no significant bactericidal activity against *Listeria*. Furthermore, Ply2741 displays better bactericidal activity against *E. faecalis* and *S. pneumoniae*, while PlySs2 demonstrates less efficacy against *E. faecalis* compared to *S. aureus* and *Streptococcus* [57,58]. These results suggest potential therapeutic benefits in treating infections caused by VRE. EDTA usually plays the role of chelating metal ions and is able to affect the lytic activity of endolysins with CHAP domain [27]. PlySs2 showed a significant decrease in lytic activity after the addition of 4 μM EDTA [56], while Ply2741 was able to tolerate 10 μM EDTA. This result indicates that differences in CHAP domains result in varying metal ion-binding capacities between Ply2741 and PlySs2, which in turn affecting the dependence of endolysins on metal ions. Although the lytic spectrum of Ply2741 is not as extensive as PlySs2, it possesses a rare lytic profile among the other lysins. Consequently, the characterization of Ply2741 enriches the endolysin database and provides additional options comparable to PlySs2 for combating multidrug-resistant infections.

Treatment with Ply2741 significantly improved mouse survival rates in a dose-dependent manner, with the 400 μg dose showing no significant difference in protective efficacy compared to ampicillin. However, although the *in vivo* therapeutic effect of Ply2741 was not as strong as that of AVPL and Ply1228 [54,59], its broad-spectrum bactericidal activity against various bacterial species offers greater potential for further modification. The chimeric lysin ClyQ, compared to the parental lysin LysGH15, not only exhibited significantly enhanced bactericidal activity but also expanded its lytic spectrum [39]. Additionally, the combination of daptomycin and lysin Cpl-1 demonstrated higher protective efficacy against *S. pneumoniae* infection compared to each agent used individually [60]. Therefore, the development of chimeric lysins and the synergistic application of Ply2741 with antibiotics are worth investigation in future studies. Furthermore, treatment with Ply2741 via various routes of administration can significantly reduce bacterial loads and inflammatory cytokines in organs and provide substantial protection. This therapeutic route provides additional options for the clinical application of endolysins.

Considering the lytic activity of Ply2741, investigation of its catalytic mechanism is necessary. Sequence and structural analyses reveal a classic “Cys-His-Asn” catalytic triad within the CHAP domain of Ply2741. This catalytic triad structure is a common active site in various metal-independent enzymes, including cysteine proteinases papain [61]. Cysteine proteinases typically have a conserved structural core consisting of an α-helix followed by antiparallel β-strands, with the active site located between the helix [62]. In this structure, the classic catalytic center comprises cysteine and histidine. The thiol group of cysteine is positioned at the N-terminus of the core α-helix and acts as a nucleophile during the catalytic process, while histidine functions as a base to form a reactive thiolate-imidazolium ion pair [63]. The interaction between the thiolate anion of cysteine and the imidazolium ion is crucial for the catalytic function [64]. Additionally, the amide oxygen of asparagine forms a hydrogen bond with histidine, allowing the imidazole ring to rotate without disrupting the hydrogen bond, thereby stabilizing the entire catalytic system [65]. In light of this, we propose that the catalytic triad in Ply2741 has a similar mechanism. Cys30 serves as a nucleophile attacking the carbonyl carbon of the bacterial peptidoglycan amide bond, forming a covalent acyl-enzyme intermediate. His97 can form an imidazolium ion pair to enhance the cysteine nucleophilic attacking ability. Asn115 stabilizes the system by forming hydrogen bonds with His97. The mutation of His97 results in the loss of hydrogen bonding interactions with cysteine and asparagine, which explains the higher reduction in thermal stability observed in the H97A mutant [66].

Interestingly, a conserved amino acid residue, Gln29, located near the catalytic triad, also affects the bactericidal activity of Ply2741. To our knowledge, this is the first report of a conserved residue near the catalytic center being related to lytic activity of endolysin. Although Gu et al. identified a conserved E134 residue near the catalytic center in LysGH15 and defined it along with “Cys-His-Glu-Asn” as a catalytic quartet, the lytic activity did not completely disappear after E134 mutation [27]. Multiple sequence alignments reveal that a conserved Gln residue is present in nearly all CHAP endolysins, but its function has not been reported previously. Glutamine in PlyPy and PlySs2, which are highly similar in structure and sequence to Ply2741CHAP, may have similar functions which provide a theoretical basis for further engineering of endolysins. However, the existing evidence does not elucidate the specific role of Gln29 in catalytic activity of Ply2741. We speculate that Gln29 may play a role in stabilizing the catalytic triad. Furthermore, although some studies suggest that Gln residues in certain cysteine proteases might stabilize tetrahedral by forming the oxyanion hole during catalysis [67], the position of Gln29 in Ply2741 is not consistent with cysteine proteases, indicating the need for further research to verify its potential role in the catalytic mechanism.

The Ply2741CHAP shares a sequence similarity with PlyPy, which demonstrates activity only against *Streptococcus* [68]. Therefore, the extensive-binding capabilities of Ply2741CBD are of interest. Previous studies have confirmed that endolysins exert bactericidal effects by destroying peptidoglycans [68,69]. Additionally, Eugster et al. found that the CBDs of Ply118, Ply511, and PlyP40 specifically interact with the peptidoglycan backbone of *Listeria monocytogenes* [70]. Gonzalez-Delgado et al. further elucidated the structural basis of the binding of Lysostaphin SH3b to *Staphylococcus* peptidoglycan using NMR [30]. However, it is noteworthy that other studies suggest that SH3b domains may interact with the bacterial wall teichoic acid (WTA) rather than peptidoglycan. Shen et al. discovered that the *Listeria* endolysin CBD500 specifically recognizes WTA on the bacterial surface. Structural analysis of CBD500 revealed that the binding cavity formed by β-barrel and pseudo-symmetric SH3b-like repeats serves as the structural basis for WTA recognition, a feature that is absent in Ply2741SH3b [71]. Conversely, Ply2741SH3b shares a high structural similarity with Lysostaphin SH3b, which binds to the peptidoglycan. Therefore, we explored the mechanism of the Ply2741CBD-PG interaction. However, the potential interaction between Ply2741SH3b and WTA, and its underlying mechanism, remain worthy of further investigation.

Ply2741 exhibits broad-spectrum bactericidal activity and shares sequence and structural similarity with the Lysostaphin SH3b. Although the streptococcal endolysin PlySs2, which also has broad-spectrum activity, does not bind to *Staphylococcus*, this could be due to limitations in detection sensitivity and resolution [58]. FACS experiments have demonstrated that Ply2741CBD can significantly bind to *S. aureus*. Given the composition of *Streptococcus* and *Staphylococcus* peptidoglycan, L-alanyl-D-isoglutamine, P4, P4-2A, and P4-G5 of peptidoglycan were selected as candidate substrate molecules. Molecular docking results revealed that all candidate molecules were located in the same pocket region, implying that this region may act as the key binding region of Ply2741. MD analysis revealed an extreme instability in the complex of CBD and substrate P4-2A and P4-G5. Notably, the binding pocket of CBD predominantly interacted with the P4 segment of the P4-2A and P4-G5 molecule. In addition, the substrate molecule P4 contains L-alanyl-D-isoglutamine and compared with L-alanyl-D-isoglutamine, substrate P4 also exhibited instability during the process. Ji et al. determined the crystal structure of the Ly7917 SH3b-substrate complex using X-ray diffraction (PDB id: 5d76). Their findings revealed that Ly7917 SH3b specifically binds to L-alanyl-D-isoglutamine through the residues A176 and R180, which constitute key substrate-binding sites [30,72]. Considering their structural similarity, L-alanyl-D-isoglutamine was identified as the substrate of Ply2741CBD. However, it is challenging to determine the binding affinity of CBD with L-alanyl-D-isoglutamine through experiments, likely because of the very weak binding affinity of CBD to the substrate, as found in previous studies [27,73,74]. Analysis of the interaction between L-alanyl-D-isoglutamine and Ply2741CBD revealed the intermolecular interactions between L-alanyl-D-isoglutamine and the residues Ser185, Asn187, Arg189, and Trp250 around the binding pocket. Except for the π–sigma interaction with W250, CBD interacted with the other three amino acids via hydrogen bond interactions. FACS and turbidity reduction assay results showed that the mutation of R189 and W250 significantly affected protein-binding activity. Interestingly, the binding ability of the S185 and N187 mutations and their impact on mature protein activity were not significant. Although mutations at R189 and W250 significantly reduced protein activity, they were not completely abolished. This phenomenon is attributed to the fact that the mutation of a single binding site does not completely lose the binding ability, and the additional-binding site may be compensated, aligning with the findings of Gonzalez-Delgado et al. in the study of lysostaphin SH3b [30].

In summary, we identified an endolysin, Ply2741, which demonstrated significant broad-spectrum bactericidal efficacy against multidrug-resistant gram-positive bacteria *in vitro* and *in vivo*. Through structural analysis of Ply2741 catalytic and cell wall binding domains, we elucidated the mechanism of the “Cys-His-Asn” catalytic triad in the catalytic domain of Ply2741 and identified that the conserved residue Gln29 also affects the lytic activity. Additionally, we systematically characterized the key residues and interaction modes of the Ply2741 CBD involved in binding to bacterial peptidoglycan.

## Supplementary Material

Figure S1.tif

ARRIVE_Author_Checklist.pdf

Figure S3.tif

Figure S5.tif

Figure S2.tif

Figure S4.tif

Supplemental_table.docx

Figure S6.tif

## Data Availability

The data that support the findings of this study are openly available in Zenodo at https://doi.org/10.5281/zenodo.14292097.
