# Supplementary figures and images for: Structural and functional analysis reveals the catalytic mechanism and substrate binding mode of the broad-spectrum endolysin Ply2741
Source: Virulence. 2025 Jan 14;16(1):2449025. doi: 10.1080/21505594.2024.2449025 (PMC11740692; doi:10.1080/21505594.2024.2449025)

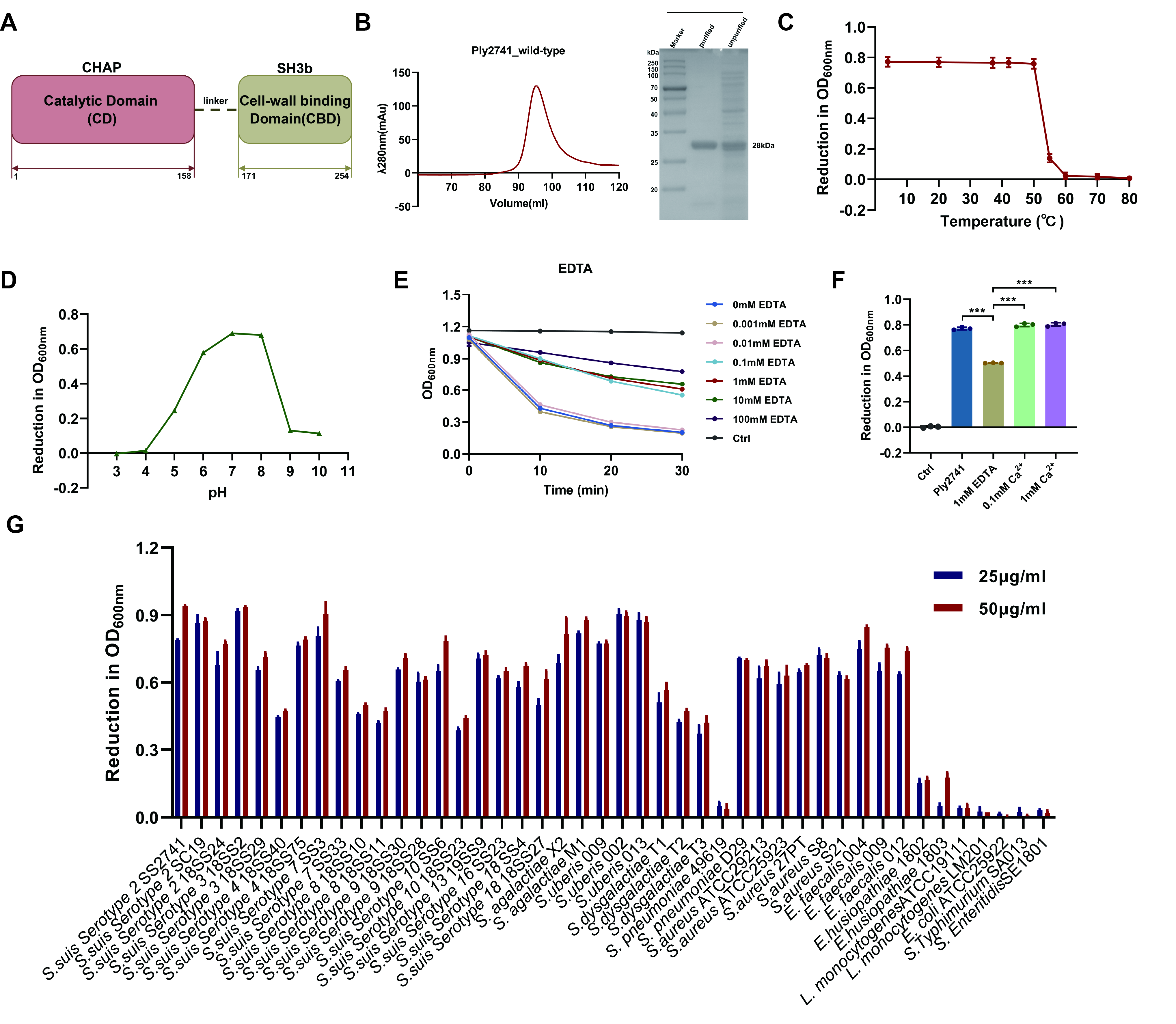

Supplement: Figure S1.tif [file KVIR_A_2449025_SM9222.tif]

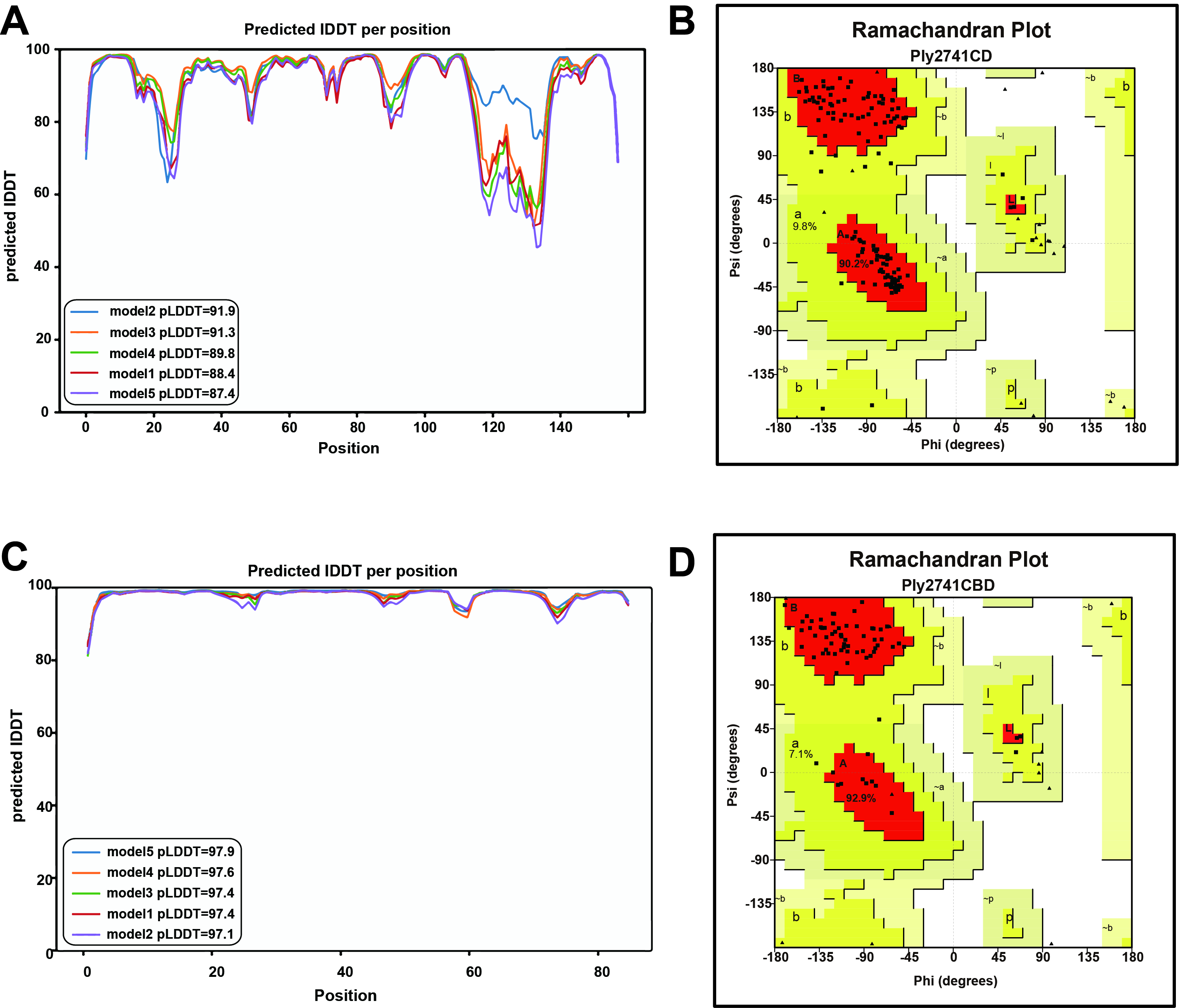

Supplement: Figure S3.tif [file KVIR_A_2449025_SM9220.tif]

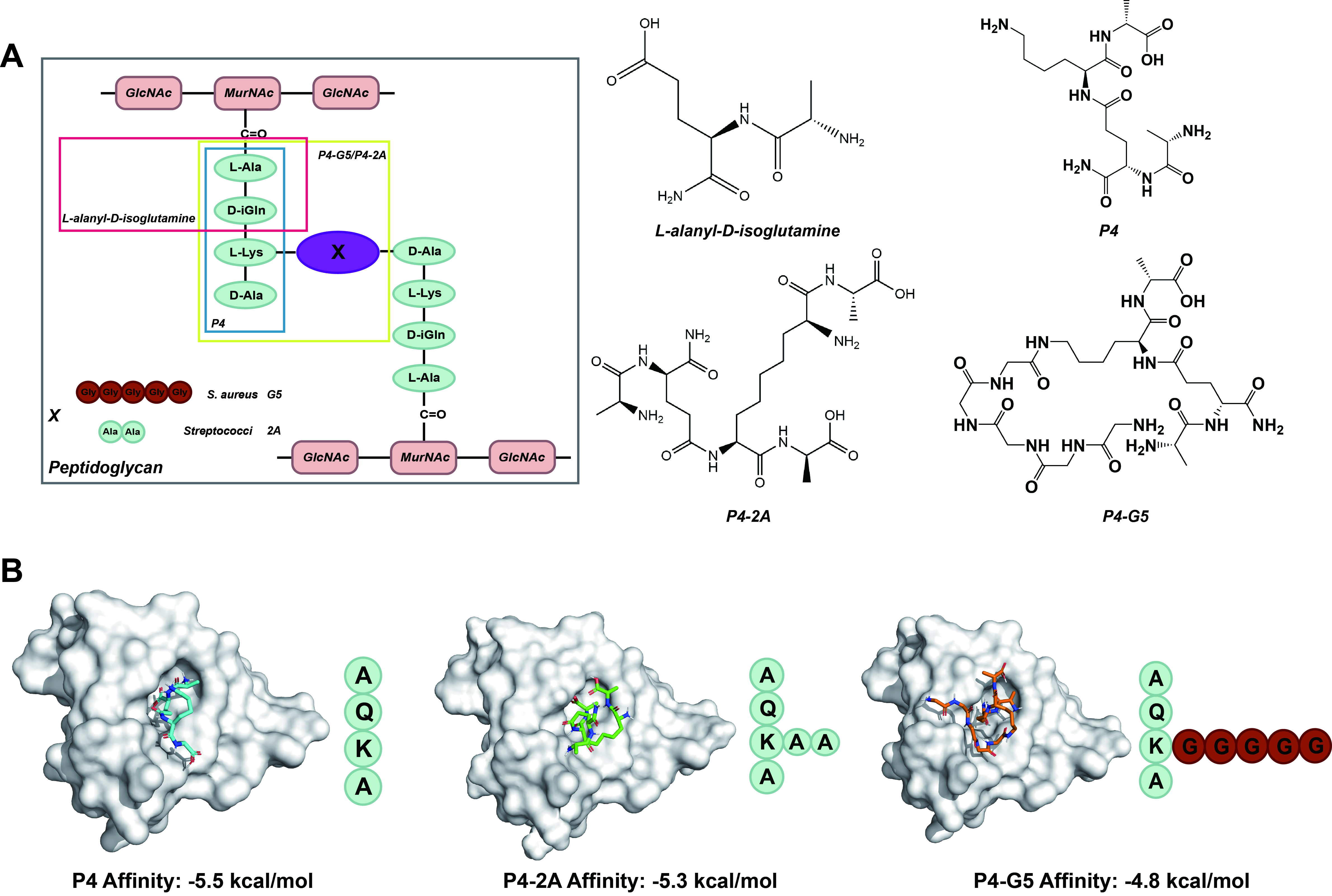

Supplement: Figure S5.tif [file KVIR_A_2449025_SM9219.tif]

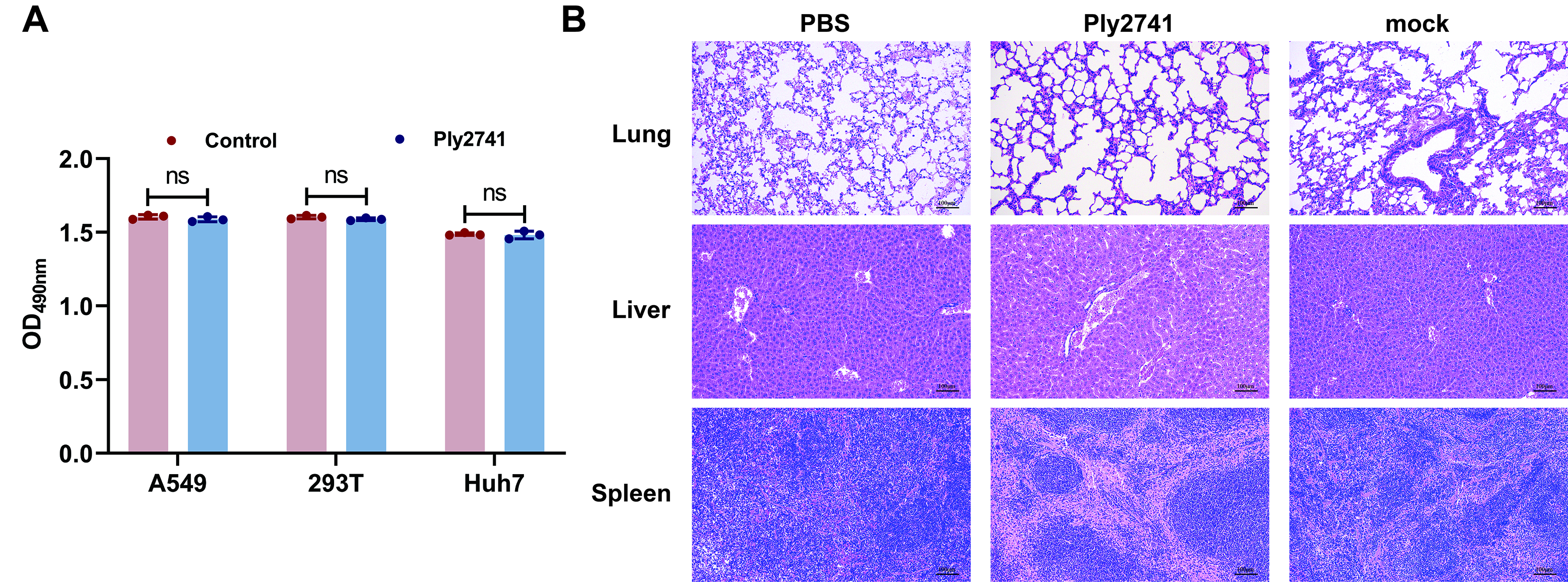

Supplement: Figure S2.tif [file KVIR_A_2449025_SM9218.tif]

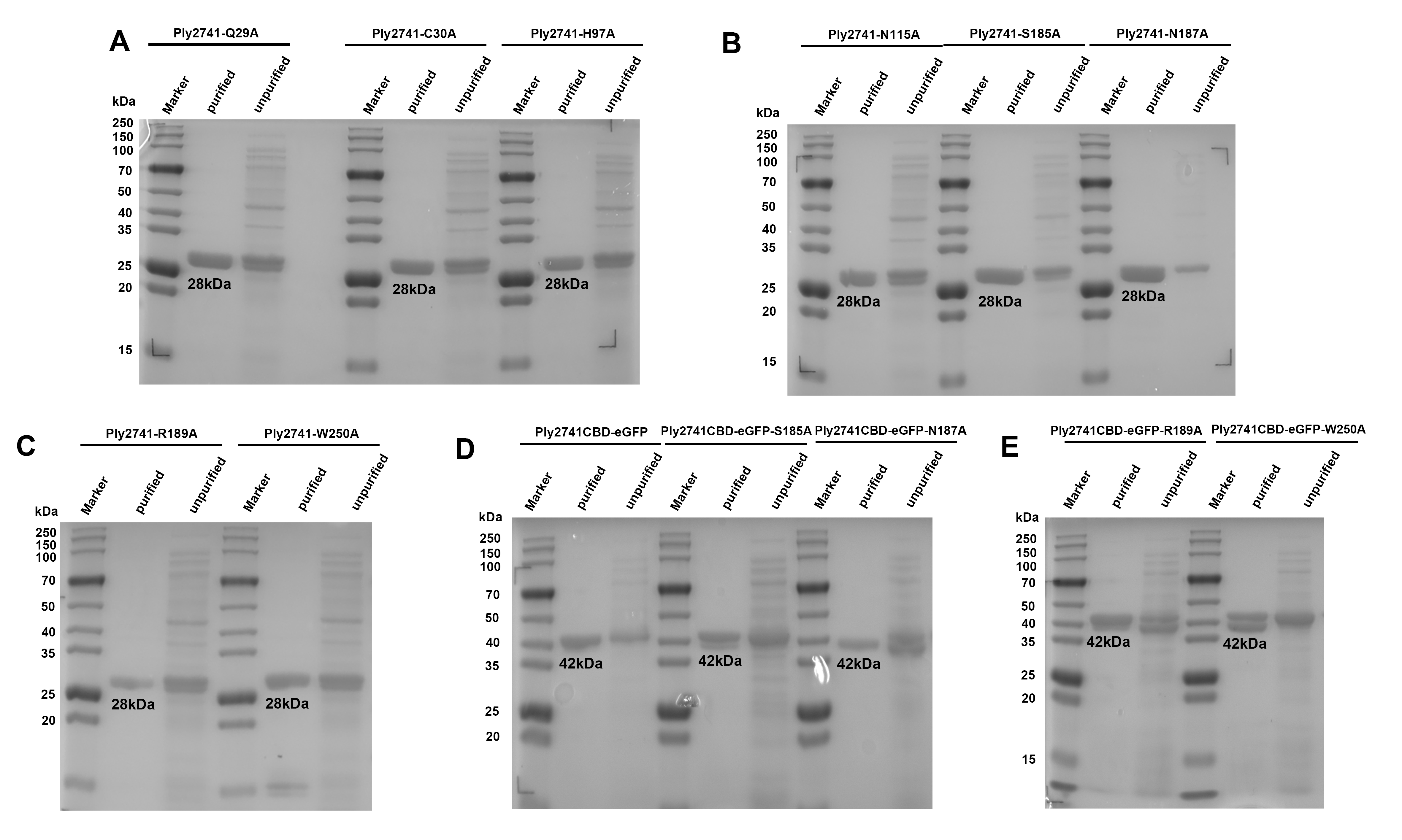

Supplement: Figure S4.tif [file KVIR_A_2449025_SM9217.tif]

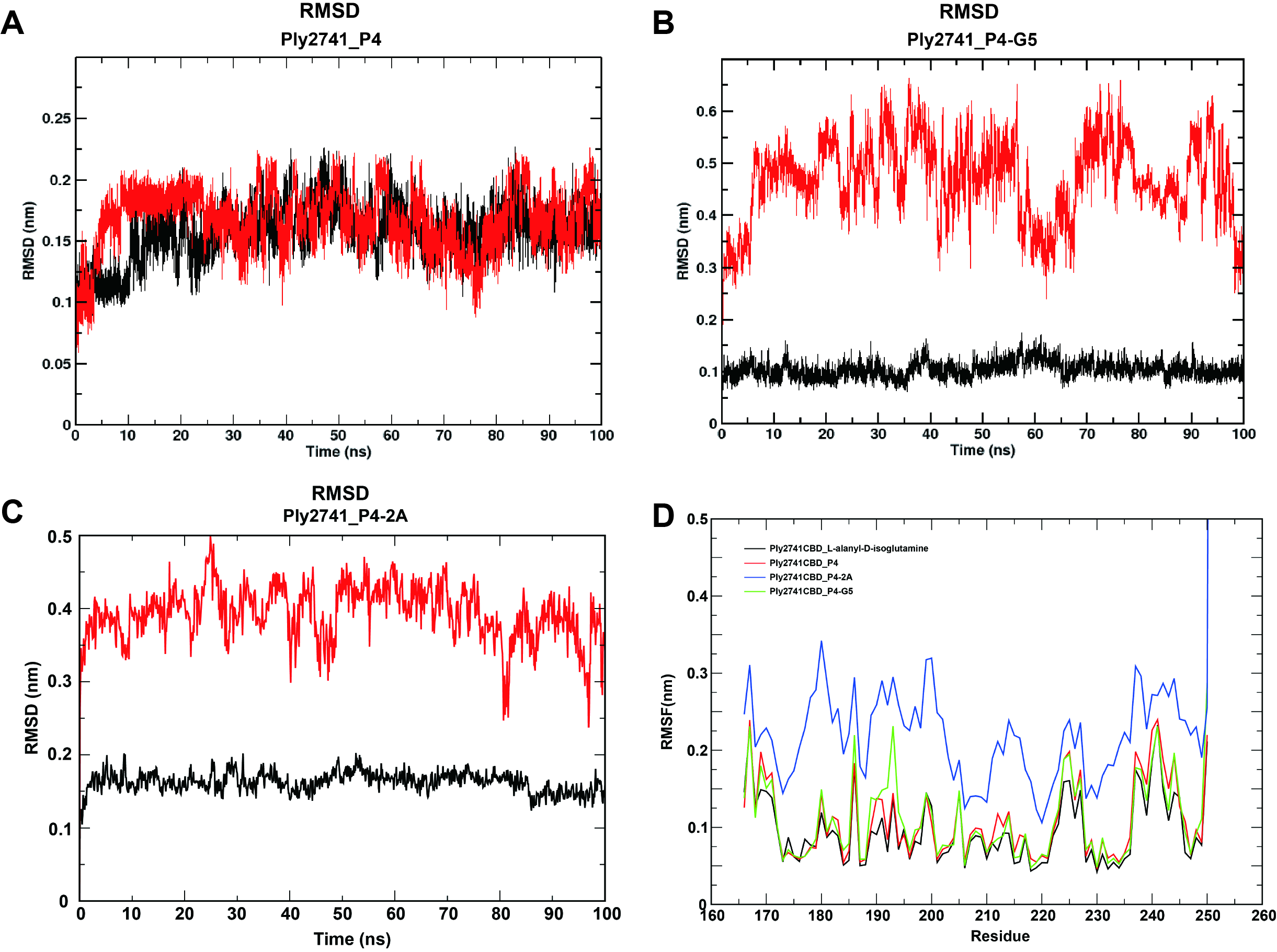

Supplement: Figure S6.tif [file KVIR_A_2449025_SM9215.tif]
