# Supplementary material for: Structural and functional analysis reveals the catalytic mechanism and substrate binding mode of the broad-spectrum endolysin Ply2741
Source: Virulence. 2025 Jan 14;16(1):2449025. doi: 10.1080/21505594.2024.2449025 (PMC11740692; doi:10.1080/21505594.2024.2449025)
Supplement: Supplemental_table.docx [file KVIR_A_2449025_SM9216.docx]

**Table S1.** The results of spot-test assay of Ply2741 against different genus bacteria

| **Species** | **lytic** | **Species** | **lytic** | **Species** | **lytic** | **Species** | **lytic** |
| --- | --- | --- | --- | --- | --- | --- | --- |
| *S. suis* SS1 | + | *S. suis* SS26 | + | *S. suis* SSLab24 | + | *S. suis* 19SS 12 | - |
| *S. suis* SS2 | - | *S. suis* SS27 | + | *S. suis* SSLab20 | - | *S. suis* 19SS 13 | - |
| *S. suis* SS3 | + | *S. suis* SS28 | + | *S. suis* SSLab29 | + | *S. suis* 19SS 14 | - |
| *S. suis* SS4 | - | *S. suis* SS29 | + | *S. suis* SSLabN0 | + | *S. suis* 19SS 15 | - |
| *S. suis* SS5 | + | *S. suis* SS30 | + | *S. suis* SSLabN1 | + | *S. suis* 19SS 16 | + |
| *S. suis* SS6 | - | *S. suis* SS31 | + | *S. suis* SSLabN2 | + | *S. suis* 19SS 17 | + |
| *S. suis* SS7 | + | *S. suis* SS32 | + | *S. suis* SSLabN3 | - | *S. suis* 19SS 18 | + |
| *S. suis* SS8 | - | *S. suis* SS33 | + | *S. suis* SSLabN4 | + | *S. suis* 19SS 19 | - |
| *S. suis* SS9 | + | *S. suis* SS34 | + | *S. suis* SSLabN5 | + | *S. suis* 19SS 20 | - |
| *S. suis* SS10 | - | *S. suis* SS35 | + | *S. suis* SSLabN6 | + | *S. suis* 19SS 21 | + |
| *S. suis* SS11 | + | *S. suis* SS36 | + | *S. suis* SSLabN9 | + | *S. suis* 19SS 22 | + |
| *S. suis* SS12 | - | *S. suis* SS37 | + | *S. suis* SSLabN11 | + | *S. suis* 19SS 23 | + |
| *S. suis* SS13 | - | *S. suis* SS38 | + | *S. suis* SSLabN15 | + | *S. suis* 19SS 24 | + |
| *S. suis* SS14 | - | *S. suis* SS40 | + | *S. suis* 19SS 1 | - | *S. suis* 19SS 25 | + |
| *S. suis* SS15 | + | *S. suis* SS41 | + | *S. suis* 19SS 2 | + | *S. suis* 19SS 26 | + |
| *S. suis* SS16 | + | *S. suis* SS42 | + | *S. suis* 19SS3 | - | *S. suis* 19SS 27 | + |
| *S. suis* SS17 | + | *S. suis* SS43 | + | *S. suis* 19SS4 | + | *S. suis* 19SS 28 | + |
| *S. suis* SS18 | + | *S. suis* SSLab26 | - | *S. suis* 19SS5 | - | *S. suis* 19SS 29 | + |
| *S. suis* SS19 | - | *S. suis* SSLab30 | + | *S. suis* 19SS6 | + | *S. suis* 19SS 30 | + |
| *S. suis* SS20 | + | *S. suis* SSLab6 | + | *S. suis* 19SS7 | - | *S. suis* SC19 | + |
| *S. suis* SS21 | + | *S. suis* SSLab15 | + | *S. suis* 19SS 8 | - | *S. suis* 18SS 1 | - |
| *S. suis* SS23 | - | *S. suis* SSLab19 | - | *S. suis* 19SS9 | - | *S. suis* 18SS 2 | + |
| *S. suis* SS24 | + | *S. suis* SSLab12 | + | *S. suis* 19SS10 | + | *S. suis* 18SS 3 | + |
| *S. suis* SS25 | - | *S. suis* SSLab22 | - | *S. suis* 19SS 11 | - | *S. suis* 18SS 4 | + |
| *S. suis* 18SS 5 | - | *S. suis* 18SS 30 | + | *S. suis* 18SS 55 | + | *S. suis* 18SS 80 | + |
| *S. suis* 18SS 6 | - | *S. suis* 18SS 31 | + | *S. suis* 18SS 56 | + | *S. suis* 18SS 81 | + |
| *S. suis* 18SS 7 | + | *S. suis* 18SS 32 | - | *S. suis* 18SS 57 | + | *S. suis* 18SS 82 | - |
| *S. suis* 18SS 8 | - | *S. suis* 18SS 33 | + | *S. suis* 18SS 58 | + | *S. suis* 18SS 83 | - |
| *S. suis* 18SS 9 | + | *S. suis* 18SS 34 | + | *S. suis* 18SS 59 | + | *S. suis* 18SS 84 | + |
| *S. suis* 18SS 10 | - | *S. suis* 18SS 35 | + | *S. suis* 18SS 60 | - | *S. suis* 18SS 85 | + |
| *S. suis* 18SS 11 | - | *S. suis* 18SS 36 | - | *S. suis* 18SS 61 | + | *S. suis* 18SS 86 | + |
| *S. suis* 18SS 12 | + | *S. suis* 18SS 37 | + | *S. suis* 18SS 62 | - | *S. suis* 18SS 87 | + |
| *S. suis* 18SS 13 | + | *S. suis* 18SS 38 | - | *S. suis* 18SS 63 | - | *S. suis* 18SS 88 | - |
| *S. suis* 18SS 14 | - | *S. suis* 18SS 39 | + | *S. suis* 18SS 64 | + | *S. suis* 18SS 89 | + |
| *S. suis* 18SS 15 | + | *S. suis* 18SS 40 | + | *S. suis* 18SS 65 | - | *S. suis* 18SS 90 | - |
| *S. suis* 18SS 16 | - | *S. suis* 18SS 41 | + | *S. suis* 18SS 66 | + | *S. suis* 18SS 91 | + |
| *S. suis* 18SS 17 | - | *S. suis* 18SS 42 | - | *S. suis* 18SS 67 | + | *S. uberis* ZMD006 | + |
| *S. suis* 18SS 18 | + | *S. suis* 18SS 43 | + | *S. suis* 18SS 68 | + | *S. uberis* ZMD009 | + |
| *S. suis* 18SS 19 | - | *S. suis* 18SS 44 | - | *S. suis* 18SS 69 | - | *S. uberis* ZMD013 | + |
| *S. suis* 18SS 20 | + | *S. suis* 18SS 45 | - | *S. suis* 18SS 70 | + | *S. uberis* H13 | + |
| *S. suis* 18SS 21 | + | *S. suis* 18SS 46 | + | *S. suis* 18SS 71 | + | *S. uberis* HB190 | + |
| *S. suis* 18SS 22 | + | *S. suis* 18SS 47 | + | *S. suis* 18SS 72 | + | *S. uberis* HB017 | + |
| *S. suis* 18SS 23 | + | *S. suis* 18SS 48 | - | *S. suis* 18SS 73 | - | *S. uberis* HB018 | + |
| *S. suis* 18SS 24 | + | *S. suis* 18SS 49 | + | *S. suis* 18SS 74 | - | *S. uberis* 002 | + |
| *S. suis* 18SS 25 | - | *S. suis* 18SS 50 | + | *S. suis* 18SS 75 | + | *S. uberis* 013 | + |
| *S. suis* 18SS 26 | + | *S. suis* 18SS 51 | - | *S. suis* 18SS 76 | + | *S. agalactiae* M1 | + |
| *S. suis* 18SS 27 | - | *S. suis* 18SS 52 | + | *S. suis* 18SS 77 | - | *S. agalactiae* X2 | + |
| *S. suis* 18SS 28 | + | *S. suis* 18SS 53 | - | *S. suis* 18SS 78 | + | *S. agalactiae* 5374 | + |
| *S. suis* 18SS 29 | + | *S. suis* 18SS 54 | + | *S. suis* 18SS 79 | - | *S. agalactiae* 13813 | + |
| *S. dysgalactiae* H1-1 | + | *S. aureus* ST95 | + | *S. aureus* S8 | + | *S. aureus* S25 | + |
| *S. dysgalactiae* T1 | + | *S. aureus* ST18 | + | *S. aureus* S9 | + | *S. aureus* S26 | + |
| *S. dysgalactiae* T2 | + | *S. aureus* ST76 | + | *S. aureus* S10 | + | *S. aureus* S27 | - |
| *S. dysgalactiae* T3 | - | *S. aureus* ST80 | + | *S. aureus* S11 | + | *S. aureus* S28 | - |
| *E. rhusiopathiae* 1774 | - | *S. aureus* ST39 | + | *S. aureus* S12 | + | *S. aureus* S29 | + |
| *E. rhusiopathiae* 1802 | - | *S. aureus* 785 | - | *S. aureus* S13 | + | *S. epidermidis* Z17 | + |
| *E. rhusiopathiae* 1803 | - | *S. aureus* 538 | - | *S. aureus* S14 | + | *S. epidermidis* Z21 | + |
| *S. aureus* 0116BP1A | + | *S. aureus* atcc29213 | + | *S. aureus* S15 | + | *E. faecalis* 009-2 | + |
| *S. aureus* 0116BP2A | + | *S. aureus* atcc43300 | - | *S. aureus* S16 | + | *E. faecalis* 012-2 | + |
| *S. aureus* 0414P6A | + | *S. aureus* atcc25923 | + | *S. aureus* S17 | + | *E. faecalis* 004-2 | + |
| *S. aureus* 0603P16A | + | *S. aureus* S1 | + | *S. aureus* S18 | - | *Listeria monocytogenes* ATCC19111 | - |
| *S. aureus* 27PT | + | *S. aureus* S2 | + | *S. aureus* S19 | + | *Listeria monocytogenes* LM201 | + |
| *S. aureus* 0316-M1B | + | *S. aureus* S3 | + | *S. aureus* S20 | + | *E. coli* ATCC25922 | - |
| *S. aureus* 0316-P8B | + | *S. aureus* S4 | - | *S. aureus* S21 | + | *E. coli* BL21 | - |
| *S. aureus* 0414P6A | + | *S. aureus* S5 | + | *S. aureus* S22 | + | *E. coli* DH5α | - |
| *S. aureus* 0509-m6a | + | *S. aureus* S6 | + | *S. aureus* S23 | + | *E. coli* HB10 | - |
| *S. aureus* 0509-M9A | + | *S. aureus* S7 | + | *S. aureus* S24 | + | *Salmonella* SE1081 | - |
| *Salmonella* SE1812 | - | *Salmonella* Sal013 | - |  |  |  |  |

"+"lysis; "-" unlysis (no clear inhibitory halos)

**Table S2.** Bacteria used in turbidity reduction assay

| **Genus** | **Species** | **Name** | **Source** |
| --- | --- | --- | --- |
| ***Streptococcus*** | *Streptococcus suis* | SS2741 | [1] |
|  | *Streptococcus suis* | SC19 | [2] |
|  | *Streptococcus suis* | N15 | Clinical isolate |
|  | *Streptococcus suis* | 18SS24 | Clinical isolate |
|  | *Streptococcus suis* | 18SS2 | Clinical isolate |
|  | *Streptococcus suis* | 18SS29 | Clinical isolate |
|  | *Streptococcus suis* | 18SS40 | Clinical isolate |
|  | *Streptococcus suis* | 18SS75 | Clinical isolate |
|  | *Streptococcus suis* | SS3 | Clinical isolate |
|  | *Streptococcus suis* | SS33 | Clinical isolate |
|  | *Streptococcus suis* | 18SS10 | Clinical isolate |
|  | *Streptococcus suis* | 18SS11 | Clinical isolate |
|  | *Streptococcus suis* | 18SS30 | Clinical isolate |
|  | *Streptococcus suis* | 18SS28 | Clinical isolate |
|  | *Streptococcus suis* | SS6 | Clinical isolate |
|  | *Streptococcus suis* | 18SS23 | Clinical isolate |
|  | *Streptococcus suis* | 19SS9 | Clinical isolate |
|  | *Streptococcus suis* | SS23 | Clinical isolate |
|  | *Streptococcus suis* | SS4 | Clinical isolate |
|  | *Streptococcus suis* | 18SS27 | Clinical isolate |
|  | *Streptococcus agalactiae* | ATCC13813 | Lab strain |
|  | *Streptococcus agalactiae* | X2 | Clinical isolate |
|  | *Streptococcus agalactiae* | M1 | Clinical isolate |
|  | *Streptococcus uberis* | 009 | Clinical isolate |
|  | *Streptococcus uberis* | 002 | Clinical isolate |
|  | *Streptococcus uberis* | 013 | Clinical isolate |
|  | *Streptococcus dysgalactiae* | T1 | Clinical isolate |
|  | *Streptococcus dysgalactiae* | T2 | Clinical isolate |
|  | *Streptococcus dysgalactiae* | T3 | Clinical isolate |
|  | *Streptococcus pneumoniae* | D29 | Clinical isolate |
|  | *Streptococcus pneumoniae* | 49619 | Clinical isolate |
| ***Staphylococcus*** | *Staphylococcus aureus* (MRSA) | S8 | [3] |
|  | *Staphylococcus aureus* | ATCC25923 | Lab strain |
|  | *Staphylococcus aureus* | ATCC29213 | Lab strain |
|  | *Staphylococcus aureus* (MRSA) | S21 | Clinical isolate |
|  | *Staphylococcus aureus* | 27PT | Clinical isolate |
|  | *Staphylococcus epidermidis* | Z17 | [3] |
| ***Enterococcus*** | *Enterococcus faecalis* (VRE) | 004-1 | Clinical isolate |
|  | *Enterococcus faecalis* | 009-1 | Clinical isolate |
|  | *Enterococcus faecalis* | 012-1 | Clinical isolate |
| ***Erysipelothrix*** | *Erysipelothrix rhusispathiae* | 1802 | Clinical isolate |
|  | *Erysipelothrix rhusispathiae* | 1803 | Clinical isolate |
| ***Listeria*** | *Listeria monocytogenes* | ATCC19111 | Lab strain |
|  | *Listeria monocytogenes* | LM201 | Clinical isolate |
| ***Escherichia*** | *Escherichia coil* | ATCC25922 | Lab strain |
| ***Salmonella*** | *S.* Typhimurium | SA013 | [4] |
|  | *S.* Enteritidis | SE1801 | [5] |

**Table S3.** Endolysins and their amino acid sequences involved in the phylogenetic analysis

| Endolysin | Amino acid sequence |
| --- | --- |
| Ply0180 | MSNLGLKLVQMPVPAAKYGIKCPNAMVPQWLTIHNTANNASALAEISYMNGNWNEVSYHWAVDDVQAIQAIPHNRNAWHCGDGTNGAGNRRSIGIEICYSLTPGHPKYAKAEDNGAKLAAIILHQLGWGIDRIRKHQDWSGKYCPHRILDNGNWDGFKGKVQAYLLQLQGKAVVAPQPAPKISTAQPRTASGNTGTREYAETGVFTATENIYFRNEPNLNGHTQGMYYKGESVTYDRVRVDYNGFVWISWISASTGVRRWMPIKIRKNGQTTEVWGNVK |
| Ply0183 | MSNLGLKMIQMLVPAAKYGIKCPNAMVPQWLTIHNTANNASALAEISYMNGNWNEVSYHWAVDDEQAIQAIPHNRNAWHCGDGTNGTGNRKSIGIEICYSLTPGHPKYAKAEDNGAKLAAIILHQLGWGVDRIRKHQDWSGKYCPHRILDNGNWDCRSLATTILVYHFL |
| Ply0262 | MGKHLVICGHGQGRTGYDPGAVNAKLGITEAGKVRELAKLMSKYSGQQIDFITEQNVYDYRSITSIGKGYDSITELHFNAFNGSAKGTEVLIQSSLEADKEDMAILSLLSRYFQNRGIKKVDWLYNANQAASRGYTYRLVEIAFIDNEQDMAIFENKKEDIARGLVSAITGVEVKTIVPSTPSSTVGSSGTPSKPVYLVGDSLRVLPHATHYQTGQKIANWVKGRTYKILQVKNIHQSNSKRAYLLDGIKSWVLEQDVEGTTKGHSEQTYQAQKGDTYYGIARKFGLTVDALLAVNGLKKSDILKVGQTLKVNTASRTTTAIPTSVASRVVASALSKVGQKVTVPSNPYGGQCVALVDKIVQELTDKNMSYTNAIDCLKKAKSNGFQVIYDAWGVNPKAGDFYVIQTDGLVYGHIGVCVMDSDGKSIDGVEQNIDGYSDHNKNGINDQLEIGGGGITRRVKRQWMTDGSLYDSTGTVKLGKVVGWFRIS |
| Ply0859 | MTTVFEVVNFAKDLANRGQGVDYDGWYGNQCVDLPNWISGKFFGKALWGNAIDLIKSAKQHGFEVHYMPTSERPRPGAIFVKNYWANDGGNYGHTGLIIGVSGNTVQTIEQNLVGNLSVGGPAQYSSQQISNLVGWFYPPYSDSTAVATQSSSGNLGKVKDEQGTMTVKVSLLNVRDKPGLDGKVVATYTNSEQFNYDSVYIADGYIWVSYVSRSGVRRYVAAGEESNRRNVVPYGIFK |
| Ply0899 | MGKHLVICGHGQGRTGYDPGAVNAKLGITEAGKVRELAKLMSKYSGQQIDFITEQNVYDYRSITSIGKGYDSITELHFNAFNGSAKGTEVLIQSSLEADKEDMAILSLLSRYFQNRGIKKVDWLYNANQAASRGYTYRLVEIAFIDNEHDMAIFETKKEDIAKGLVAAITGVEVKTIVPSPPNSIAGSSGTPTKPIYLVGDSLRVLPHATHYQTGQKIANWVKGRTYKILQVKNVHQSNSKRAYLLDGIKSWVLEQDVEGTTKGHSEQTYQAQKGDTYYGIARKFGLSVDALLAVNSLKKTDILRVGQTLKVNAVSRTTTAIPTSVASRVVASALSKVGQKVTVPSNPYGGQCVALVDKIVQELTDKNMSYTNAIDCLKKAKSNGFQVIYDAWGVNPKAGDFYVIQTDGLVYGHIGVCVTDSDGKSIDGVEQNIDGYSDHNRNGINDQLEIGGGGITRRVKRQWMADGSLYDSTGTVKLGKVVGWFRIS |
| Ply1369 | MTTVNEVVNFAKDLANRGQGVDYDGWYGNQCVDLPNWICGKFFGKALWGNAIDLIKSAEQHGFEVHYMPTSESPRPGAIFVKNYWAGDGINYGHTGLILGVNGNTVQTIEQNLVGNLSVGGPAQYSSQQISNLVGWFYPPYSDSTAVATQSSSGNLGKVKDEQGTMTVKVSLLNVRDKPGLDGKVVATYTYGEQFNYDSVYIADGYIWVSYVSRSGVRRYVAAGEESNRRNVVPYGTFK |
| Ply1416 | MTTVNEALNNVRAQVGSGVSVGNGECYALASWYERMISPDATVGLGAGVGWVSGAIGDTISAKNIGSSYNWQANGWTVSTSGAFVPGQILTLGATATNQYGHVVIVEAVNGDQLTILEQNMYGKRYPTRNYYSAASYRQQVVHYITPPGTVAQTAPNMAGARTYRETGTMTVTVDAINVRRTPNTSGQIVAVYKSGESFDYDTVIIDVNGYVWVSYIGSSGIRNYVATGATKDGKRFGEAWGTFK |
| Ply2233 | MSNLGLKMIQMPVPAAKYGIKCPNAMVPQWLTIHNTANNASALAEISYMNGNWNEVSYHWAVDDVQAIQAIAHNRNAWHCGDGTNGTGNRKSIGIEICYSLTPGHPKYAKAEDNGAKLAAIILHQLGWGVDRIRKHQDWSGKYCPHRILDNGNWEGFKGKVQAYLLQLQGKAVVAPQPAPKVATAQPRTASVNAGAREYAETGVFTATEDIYFRNEPNLNGRTQGMYYKGESVTYDRVRVDYNGFVWISWISASTGIRRWMPIKVRKNGQTTEVWGNVK |
| Ply0217 | MGKHLVICGHGQGRTGYDPGAVNAKLGITEAGKVRELAKIMSKYSGQQIDFITEQNVYDYRSITSIGKGYDSITELHFNAFNGSAKGTEVLIQSSLEADKEDMAILSLLSRYFQNRGIKKVDWLYNANQAASRGYTYRLVEIAFIDNEQDMAIFEKKKEDIARGLVSAITGVEVKTIVPSPPSSTVGSSGTPSKPIYLVGDSLRVLAHATHYQTGQKIANWVKGRTYKILQVKNVHQSNSKRAYLLDGIKSWVLEQDVEATTKGHSEQTYQAQKGDTYYGIARKFGLTVDALLAVNGLKKTDILRVGQTLKVNSASRTTTTIPTSVASRVVASALSKVGQKVTVPSNPYGGQCVALVDKIVQELTDKNMSYTNAIDCLKKAKSNGFQVIYDAWGVNPKAGDFYVIQTDGLVYGHIGVCVSDSDGKSIDGVEQNIDGYSDHNKNGINDQLEIGGGGITRRVKRQWMADGSLYDSTGTVKLGKVVGWFRIS |
| Ply2010 | MTTANEVVQFFVNLANAGMGVDKDGMFGTQCADVPSYTAKHWFGVDLWGNAIDLINSAAAVGWEVHYMPTDANPQAGDFFVSNAFFGGVNYGHTGIVIADSDGYTMQTVEQNIDGNWDALEVGGPARFNERDFSNVVGWFRPPYADGGNTPITQPTSNEIELIPETGTFTVGDSPINVRRAPNLSGEIVAVYKPGQSVKYDSKGSANGYRWISYIGGSGLRNYMAIGQTDEAGNRISLWGTIE |
| Ply1070 | MSNLGLKMIQMPVPAAKYGIKCPNAMVPQWLTIHNTANNASALAEISYMNGNWNEVSYHWAVDDVQAIQAIAHNRNAWHCGDGLGPGNLTSIGIEICHSLTPGNPKYAKAEDNGAKLAAIILHQMGWGVDRIRKHQDWSGKYCPHRILDNGNWDGFKGKVQVYLLQLQGKAVVAPQPAPKVATAQPRTASVNTGAREYAETGVFTATEDIYFRNEPNLNGRTQGMYYKGESVTYDRVRVDYNGFVWISWISASTGIRRWMPIKVRKNGQTTEVWGNVK |
| Ply1261 | MGKHLVICGHGQGRTGYDPGAVNTKLGITEAGKVRELAKLMSKYSGQQIDFITEQNVYDFRSITSIGKGYDSITELHFNAFNGSAKGTEVLIQSSLEADKEDMGILSLLSRYFQNRGIKKVDWLYNANQAASRGYTYRLVEIAFIDNEQDMAIFENKKEDIARGLVSAITGVEVKTIVPSTPSSTAGSSGTLSKPIYLVGDSLRVLPRATHYQTGQKVANWVKGRTYKILQVKNVHQSNSKRAYLLDGIKSWVLEQDVEGTTKGHSEQTYQAQKGDTYYGIARKFGLTVDVLLAVNGLKKTDILRVGQTLKVNAASRTTTAIPTSVASRVVASALSKVGQKVTVPSNPYGGQCVALVDKIVQELTDKNMSYTNAIDCLKKAKSNGFQVMYDAWGVNPKAGDFYVIQTDGLVYGHIGVCVTDSDGKSIDGVEQNIDGYSDHNKNGINDQLEIGGGGITRRVKRQWMADGSLYDSTGTVKLGKVVGWFRIS |
| Ply1358 | MGKHLVICGHGQGRTGYDPGAVNAKLGITEAGKVRELAKLMAKYSGQQIDFITEQNVYDYRSITSIGKGYDSITELHFNAFNGSAKGTEVLIQSSLEADKEDMAILSLLSRYFQYRGIKKVDWLYNANQAASRGYTYRLVEIAFIDNEQDMAIFENKKEDIARGLVSAITGVEVKTIVPSPPSSTVGSSGTPSKPIYLVGDSLRVLPHATHYQTGQKIANWVKGRTYKIIQVKNVHQSNSKRAYLLDGIKSWVLEQDVEGTTKGHSEQTYQAQKGDTYYGIARKFGLSVDALLALNGLKKTDILRVGQTLKVNAASRTTTAIPTSVASRVVASALSKVGQKVTVPSNPYGGQCVALVDKIVQELTDKNMSYTNAIDCLKKAKSNGFQVIYDAWGVNPKAGDFYVIQTDGLVYGHIGVCVTDSDGKSIDGVEQNIDGYSDHNKNGINDQLEIGGGGITRRVKRQWMADGSLYDSTRTVKLGKVVGWFRIS |
| Ply0081 | MGKHLVICGHGQGRTGYDPGAVNAKLGITEAGKVRELAKIMSKYSGQQIDFITEQNVYDYRSISSIGKGYDSITELHFNAFNGSAKGTEVLIQSSLEADKEDMAILSLLSRYFQNRGIKKVDWLYNANQAASRGYTYRLVEIAFIDNEQDMAIFENKKEDIAKGLVSAITGVEVKTIVPSTPSSTAGSSGTPSKPIYLLGDSLRVLPHATHYQTGQKIANWVKGRTYKILQVKNVHQSNSKRAYLLDGIKSWVLEQDVEGTTKGHSEQTYQAQKGDTYYGIARKFGLTVDALLAVNGLKKTDILRVGQTLKVNAASRTTTAIPTSVASRVVASALSKVGQKVTVPSNPYGGQCVALVDKIVQELTDKNMSYTNAIDCLKKAKSNGFQVIYDAWGVNPKAGDFYVIQTDGLVYGHIGVCVTDSDGKSIDGVEQNIDGYSDHNRNGINDQLEIGGGGITRRVKRQWMADGSLYDSTGTVKLGKVVGWFRIS |
| Ply2795 | MTTVNEVVNFAKDLANRGQGVDYDGWYGKQCVDLPNWICGKFFGKPLWGNAIDLIKSAKQHDFEVHYMPTSERPRPGAIFVKNYWAGDGINYGHTGLIIGVSGNTVQTIEQNLVGNLSVGGPAQYASQQISNLVGWFYPPYSDSTAVATQSNSGNLGKVKDEQGTMTVKVSLLNVRDKPGLDGKIVATYTYGEQFNYDSVYIADGYIWVSYISRSGVRRYVAAGEESNRRNVVPYGTFK |
| Ply2481 | MGKHLVICGHGQGRTGYDPGAVNAKLGITEAGKVRELAKLMSKYSGQQIDFITEQNVYDYRSITSIGKGYDSITELHFNAFNGSAKGTEVLIQSSLEADKEDMAILSLLSRYFQNRGIKKVDWLYNANQAASRGYTYRLVEIAFIDNEQDMAIFENKKEDIARGLVSAIIGVEVKTIVPSTPSSTVGSSGTPSKPIYLVGDSLRVLPHATHYQTGQKIANWVKGRTYKILQVKNVHQSNSKRAYLLDGIKSWVLEQDLEGTTKGHSEQTYQAQKGDTYYGIARKFGLTVDALLAVNSLKKMDILRVGQTLKVNAASRTTTAIPTSVASRVVASALSKVGQKVTVPSNPYGGQCVALVDKIVQELTDKNMSYTNAIDCLKKAKSNGFQVIYDAWGVNPKAGDFYVIQTDGLVYGHIGVCVSDSDGKSIDGVEQNIDGYSDHNKNGINDQLEIGGGGITRRVKRQWMADGSLYDSTGTVKLGKVVGWFRIS |
| Ply1248 | MGKHLVICGHGQGRTGYDSGAVNTKLGITEAGKVRELAKIMSKYSGQQIDFITEQNVYDYRSITSIGKGYDSITELHFNAFNGSAKGTEVLIQSSLEADKEDMAILSLLSRYFQNRGIKKVDWLYNANQAASRGYTYRLVEIAFIDNEQDMAIYENKKEDIAKGLVSAITGVEVKTIVPSTPSSTAGGSGTPSKPIYLVGDSLRVLPHATHYQTGQKIANWVKGRTYKILQVKNVHQSNSKRAYLLDGIKSWVLEQDVEGTTKGHSEHTYQAQKGDTYYGIARKFGLTVDALLAVNGLKKTDILRVGQTLKVNAASRTTTAIPTSVASRVVASALSKVGQKVTVPSNPYGGQCVALVDKIVQELTDKNMSYTNAIDCLKKAKSNGFQVIYDAWGVNPKAGDFYVIQTDGLVYGHIGVCVTDSDGKSIDGVEQNIDGYSDHNKNGINDQLEIGGGGITRRVKRQWMADGSLYDSTGTVKLGKVVGWFRIS |
| Ply2741 | MTTANDVVSYSLSLVGRKVTVPTNPYGGQCVALIDHIMQHLTGGQLNMAYTNAKDCLVRAKKLGLSVVYNDTSKPDLIPQAGDFFVMQFGANDPFGHIGVCISANVNGMTTVEQNIDGYSDHNRNGINDQLEIGGGGITRKHQRDYSNVIGWFRLNYSSQSTGPKPATGSRRYKESGVFTVTVDSINVRRAPNTSGQIVATYKKGQSIKYDEVVIDVDGFVWISYIGGSGKRNYVATGATKDGKRFGPAWGTFK |
| Ply2761 | MGKHLVICGHGQGRTGYDPGAVNAKLGITEAGKVRELAKSMSKYSGQQIDFITEQNVYDYRSITSIGKGYDSITELHFNAFNGSAKGTEVLIQSSLEADKEDMAILSLLSRYFQNRGIKKVDWLYNANQAASRGYTYRLVEIAFIDNEHDMAIFETKKEEIAKGLVSAITGVEVKTIVPSTPSSTVGSSGTPSKPIYLVGDSLRVLPHATHYQTGQKIANWVKGRTYKILQVKNVHQSNSKRAYLLDGIKSWVLEQDVEGTTKGHSEQTYQAQKGDTYYGIARKFGLTVDALLAVNGLKKTDILRVGQTLKVNAASRTTTAIPTSVASRVVAYALSKVGQKVTVPSNPYGGQCVALVDKIVQELTDKNMSYTNAIDCLKKAKSNGFQVIYDAWGVNPKAGDFYVIQTDGLVYGHIGVCVTDSDGKSIDGVEQNIDGYSDHNKNGINDQLEIGGGGITRRLKRQWMADGSLYDSTGTVKLGKVVGWFRIS |
| Ply0796 | MGKHLVICGHGQGRTGYDPGAVNAKLGITEAGKVRELAKFMSKYSGQQIDFITEQNVYDYRSITSIGKGYDSITELHFNAFNGRAKGTEVLIQSSLEADKEDMAILSLLSRYFQNRGIKKVDWLYNANQAASRGYTYRLVEIAFIDNEHDMAIFENKKEDIARSLVSAITGVEVKAIVPSPPSSTVGSSGTPSKSIYLIGDSLRVLPHATHYQTGQKIANWVKGRTYKILQVKNVHQSNSKRAYLLDGIKSWVLEQDVEGTTKGHSEQTYQAQKGDTYYGIARKFGLSVDTLLVVNGLKKSDILKVGQTLKVNAASRTTTAIPTSVANRVVASALSKVGQKVTVPSNPYGGQCVALVDKIVQELTDKNMSYTNAIDCLKKAKSNGFRVIYDAWGVNPKAGDFYVIQTDGLVYGHIGVCVTDSDGKSIDGVEQNIDGYSDHNKNGINDQLEIGGGGITRRVKRQWMADGSLYDSTGTVKLGKVVGWFRI |
| Ply0148 | MGKHLVICGHGQGRTSYDPGAVNAKLGITEAGKVRELAKLMSKYSGQRIDFITEQNVYDYRSITSIGKGYDSITELHFNAFNGSAKGTEVLIQSSLEADKEDMAILSLLSRYFQNRGIKKVDWLYNANQAASRGYTYRLVEIAFIDNEQDMAIYENKKEDIAKGLVSAITGVEVKTIVPSTPSSTVGSSGTPSKPIYLVGDSLRVLPHATHYQTGQKIANWVKGRTYKILQVKNVHQSNSKRAYLLEGIKSWVLEQDVEGTTKGHSEQTYQAQKGDTYYGIARKFGSTVDALLAVNGLKKTDILRVGQTLKVNAALRTTTAIPTSVASRVVASALSKVGQKVTVPSNPYGGQCVALVDKIVQELTDKNMSYTNAIDCLKKAKSNGFQVIYDAWGVNPKAGDFYVIQTDGLVYGHIGVCVTDSDGKSIDGVEQNIDGYSDHNKNGINDQLEIGGGGITRRVKRQWMVGGSLYDSTGTVKLGKVVGWFRIS |
| Ply0659 | MGKHLVICGHGQGRIGYDPGAVNTKLGITEAGKVRELAKIMYKYSGQQIDFITEQNVYDYRSITSIGKGYDSITELHFNAFNGSAKGTEVLIQSSLEADKEDMAILSLLSRYFQNRGIKKVNWLYNANQAASRGYTYRLVEIAFIDNEQDMAIFENKKEDIAKGLVSAIKGVEVKTIVPSTPSSTAGSSGTPSKPIYLLGDSLRVLPHATHYQTGQKIANWVKGRTYKILQVKNVHQSNSKRAYLLDGIKSWVLEQDVEGTTKGHSEQTYQAQKGDTYYGIARKFGLTVDALLAVNGLKKTDILRVGQTLKVNAASRTTTAIPTSVASRVVASALSKVGQKVTVPSNPYGGQCVALVDKIVQELTDKNMSYTNAIDCLKKAKSNGFQVIYDAWGVNPKAGDFYVIQTDGLVYGHIGVCVTDSDGKSIDGVEQNIDGYSDHNKNGINDQLEIGGGGITRRVKRQWMADGSLYDSTGTVKLGKVVGWFRIS |
| Ply2203 | MTTVNEVTNFAKDLANRGQGVDYDGWYGKQCVDLPNWICGKYFGKALWGNAIDLIKSAKQHGFEVHYMPTSESPRPGAIFVKNYWAGDGINYGHTGLIIGVSDNTVQTIEQNLVGNLSVGGPAQYSSQQISNLVGWFYPPYSDSTAVATQASSGNLGKVKDEKGTMTVKVSLLNVRDKPGLDGKVVATYTNGEQFNYDSVYIADGYIWVSYVSRSGVRRYVAAGEESNRRNVVPYGTFK |

**Table S4.** Primers used in this study

| **Name** | **Primers** | **Use** |
| --- | --- | --- |
| **Ply2741 WT-KpnI-F** | GGGGTACCatgacaacagcaaatgatgtag | Construction of pCold-Ply2741 plasmid |
| **Ply2741 WT-HindIII-R** | CCAAGCTTctatttaaacgtaccccaagc |  |
| **Ply2741 Q29A-F** | TACGGTGGAgctTGTGTGGCTCTTATTGACCATATCA | Site-directed mutagenesis of Ply2741 wild type plasmid |
| **Ply2741 Q29A-R** | ACACAagcTCCACCGTAGGGGTTCGTTGGCAC |  |
| **Ply2741 C30A-F** | ACAGgctGTGGCTCTTATTGACCATATCATGC |  |
| **Ply2741 C30A-R** | TAAGAGCCACagcCTGTCCACCGTAGGGGTTCG |  |
| **Ply2741 H97A-F** | TTTTGGCgctATCGGTGTGTGTATCTCTGCTAATG |  |
| **Ply2741 H97A-R** | CACCGATagcGCCAAAAGGGTCATTTGCCCCA |  |
| **Ply2741 N115A-F** | TTGAGCAGgctATCGACGGCTATTCTGACCACA |  |
| **Ply2741 N115A-R** | GTCGATagcCTGCTCAACGGTTGTCATACCAT |  |
| **Ply2741 S185A-F** | CACAGTCGATgctATCAATGTCCGTCGTGCGC |  |
| **Ply2741 S185A-R** | TGATagcATCGACTGTGACTGTAAACACACCA |  |
| **Ply2741 N187A-F** | TTCTATCgctGTCCGTCGTGCGCCAAACACAT |  |
| **Ply2741 N187A-R** | GACGGACagcGATAGAATCGACTGTGACTGTAAACACA |  |
| **Ply2741 R189A-F** | ATCAATGTCgctCGTGCGCCAAACACATCTGG |  |
| **Ply2741 R189A-R** | GCACGagcGACATTGATAGAATCGACTGTGACTG |  |
| **Ply2741 W250A-F** | tcctgctgctggtacgtttaaatag |  |
| **Ply2741 W250A-R** | acgtaccagcagcaggaccaaaacgtttaccgtct |  |
| **Ply2741CBD-KpnI-F** | GGGGTACCaattattctagtcaatcaacagggccg | Construction of pCold-Ply2741CBD_eGFP plasmid |
| **Ply2741CBD-eGFP-R** | tgctcaccatcgatccagacgagcctcc |  |
| **Ply2741CBD-eGFP-F** | gtctggatcgatggtgagcaagggcgag |  |
| **eGFP-HindIII-R** | CCAAGCTTttagaattccttgtacagctcgtccatg |  |
| **Ply2741CBD_eGFP_S185A-F** | CACAGTCGATgctATCAATGTCCGTCGTGCGC | Site-directed mutagenesis of  Ply2741CBD_eGFP plasmid |
| **Ply2741CBD_eGFP_S185A-R** | TGATagcATCGACTGTGACTGTAAACACACCA |  |
| **Ply2741CBD_eGFP_N187A-F** | TTCTATCgctGTCCGTCGTGCGCCAAACACAT |  |
| **Ply2741CBD_eGFP_N187A-R** | GACGGACagcGATAGAATCGACTGTGACTGTAAACACA |  |
| **Ply2741CBD_eGFP_R189A-F** | ATCAATGTCgctCGTGCGCCAAACACATCTGG |  |
| **Ply2741CBD_eGFP_R189A-R** | GCACGagcGACATTGATAGAATCGACTGTGACTG |  |
| **Ply2741CBD_eGFP_W250A-F** | CTGCTgctGGTACGTTTAAAGGAGGCTCGTCT |  |
| **Ply2741CBD_eGFP_W250A-R** | AAACGTACCagcAGCAGGACCAAAACGTTTACCG |  |

**Table S5.** Physicochemical characteristic of Ply2741 analysed by Expasy

| **Items** | **Ply2741** |
| --- | --- |
| Size (bp) | 764 |
| Molecular Weight (kDa) | 28 |
| Theoretical PI | 9.25 |
| Formula | C_1216_H_1900_N_346_O_372_S_8_ |
| Instability Index | 26.96(Stable) |
| Estimated Half-life (hours) | 30 |
| Aliphatic Index | 74.37 |
| GRAVY | -0.344 |

**Table. S6 The MIC results of bacteria used in Kinetic time kill assay**

| **Strains** | **Species** | **Antibiotic (μg/ml)** | | | | | |
| --- | --- | --- | --- | --- | --- | --- | --- |
|  |  | **Ampicillin** | **Chloramphenicol** | **Erythromycin** | **Tetracycline** | **Oxacillin** | **Vancomycin** |
| N15 | *S. suis* | 4 | 16 | 16 | 4 | 4 | <0.5 |
| ATCC 13813 | *S. agalactiae* | <0.5 | 0.5 | <0.5 | <0.5 | <0.5 | <0.5 |
| S8 | *S. aureus* | 128 | 32 | 128 | 4 | 16 | 1 |
| Z17 | *S.epidermidis* | 64 | 16 | 128 | 4 | 16 | 0.5 |
| 004 | *E. faecalis* | <0.5 | 32 | 128 | 16 | 2 | 32 |
| 009 | *E. faecalis* | <0.5 | 32 | 128 | 16 | 1 | 4 |

MIC: Minimum Inhibitory Concentration

**Table S7.** Molecule docking results of Ply2741 with candidate substrate molecules

| Ply2741CBD_L-alanyl-D-isoglutamine | | Ply2741CBD-P4 | | Ply2741CBD-P4-2A | | Ply2741CBD-P4-G5 | |
| --- | --- | --- | --- | --- | --- | --- | --- |
| Mode | Affinity(kcal/mol) | Mode | Affinity(kcal/mol) | Mode | Affinity(kcal/mol) | Mode | Affinity(kcal/mol) |
| 1 | -5.2 | 1 | -5.5 | 1 | -5.3 | 1 | -4.8 |
| 2 | -4.9 | 2 | -5.4 | 2 | -5.2 | 2 | -4.5 |
| 3 | -4.8 | 3 | -5.3 | 3 | -5.2 | 3 | -4.4 |
| 4 | -4.7 | 4 | -5.2 | 4 | -5.1 | 4 | -4.4 |
| 5 | -4.7 | 5 | -4.8 | 5 | -5.1 | 5 | -4.3 |
| 6 | -4.7 | 6 | -4.6 | 6 | -5.1 | 6 | -4.2 |
| 7 | -4.7 | 7 | -4.6 | 7 | -5.0 | 7 | -4.2 |
| 8 | -4.6 | 8 | -4.5 | 8 | -5.0 | 8 | -4.2 |
| 9 | -4.5 | 9 | -4.5 | 9 | -4.9 | 9 | -4.2 |
| 10 | -4.4 | 10 | -4.5 | 10 | -4.9 | 10 | -4.2 |

**Reference**

1. Zou G, Zhou J, Xiao R, et al. Effects of Environmental and Management-Associated Factors on Prevalence and Diversity of Streptococcus suis in Clinically Healthy Pig Herds in China and the United Kingdom. Appl Environ Microbiol. 2018 Apr 15;84(8).

2. Teng L, Dong X, Zhou Y, et al. Draft Genome Sequence of Hypervirulent and Vaccine Candidate Streptococcus suis Strain SC19. Genome Announc. 2017 Jan 19;5(3).

3. Duan XC, Li XX, Li XM, et al. Exploiting Broad-Spectrum Chimeric Lysin to Cooperate with Mupirocin against Staphylococcus aureus-Induced Skin Infections and Delay the Development of Mupirocin Resistance. Microbiology Spectrum. 2023 Jun;11(3).

4. Wang S, Mirmiran SD, Li X, et al. Temperate phage influence virulence and biofilm-forming of Salmonella Typhimurium and enhance the ability to contaminate food product. Int J Food Microbiol. 2023 Aug 2;398:110223.

5. Gao D, Ji H, Wang L, et al. Fitness Trade-Offs in Phage Cocktail-Resistant Salmonella enterica Serovar Enteritidis Results in Increased Antibiotic Susceptibility and Reduced Virulence. Microbiol Spectr. 2022 Oct 26;10(5):e0291422.
